# Supplementary material for: Obesity in childhood, socioeconomic status, and completion of 12 or more school years: a prospective cohort study
Source: BMJ Open. 2021 Mar 11;11(3):e040432. doi: 10.1136/bmjopen-2020-040432 (PMC7957136; doi:10.1136/bmjopen-2020-040432)
Supplement: Supplementary data [file bmjopen-2020-040432supp001.pdf]

**S1 Table. International Classification of Diseases (ICD 10$^{th}$ revision) codes and Anatomical Therapeutic Chemical (ATC) classification system codes used.**

| Condition | ICD-10 | ATC Code |
|---|---|---|
| Anxiety/sedatives | F40.0-F40.1, F41-F42, F51.4-F51.9, F93.0-F93.2 | N05B |
| Depression/antidepressants | F32-F39, F92 | N06A |
| ADHD/ADD | F90 | N06B |
| Mental retardation | F70-F79 | |
| Genetic syndromes | | |
| Fragile X | Q99.2 | |
| Klinefelter | Q98 | |
| Laurence-Moon-Bardet-Biedl | Q87.8B | |
| Mb Down | Q90 | |
| Noonan | Q87.1E | |
| Prader-Willi | Q87.1F | |
| Silver Russell | Q87.1G | |
| Turner | Q96 | |
