# Supplementary material for: Obesity in childhood, socioeconomic status, and completion of 12 or more school years: a prospective cohort study
Source: BMJ Open. 2021 Mar 11;11(3):e040432. doi: 10.1136/bmjopen-2020-040432 (PMC7957136; doi:10.1136/bmjopen-2020-040432)
Supplement: Supplementary data [file bmjopen-2020-040432supp002.pdf]

S2 Table. Proportion of children completing ≥12 years of schooling

	Childhood obesity cohort		Comparison group		
	n	%	n	%	p-value¶
Total	2,236	56.7	13,939	74.4	<0.0001
Sex					
Girl	1,090	59.7	6,843	78.7	<0.0001
Boy	1,146	54.1	7,096	70.8	<0.0001
Migration background					
Nordic	1,695	58.4	10,974	78.1	<0.0001
Non-Nordic	541	52.2	2,965	63.4	<0.0001
ADHD/ADD					
Non-ADHD/ADD	2,032	61.1	13,528	76.5	<0.0001
ADHD/ADD	204	33.1	411	39.4	0.01
Anxiety/depression					
No anxiety/depression	1,902	61.1	12,754	77.0	<0.0001
Anxiety/depression	334	40.2	1,185	54.9	<0.0001
Parental SES					
Low	376	43.4	1,455	53.9	<0.0001
Medium-low	855	55.5	4,187	69.5	<0.0001
Medium-high	751	64.8	5,358	81.3	<0.0001
High	247	69.6	2,848	88.5	<0.0001
Maternal education					
Compulsory school	755	47.7	3,474	61.9	<0.0001
Upper secondary school	945	61.3	5,582	77.9	<0.0001
University degree	506	69.3	4,589	85.3	<0.0001
Paternal education					
Compulsory school	639	50.2	2,775	63.0	<0.0001
Upper secondary school	1,132	59.9	7,085	77.9	<0.0001
University degree	318	66.0	3,333	85.2	<0.0001
Maternal income					
Q1	614	52.3	3,327	68.1	<0.0001
Q2	750	58.6	4,391	75.2	<0.0001
Q3	542	58.2	3,534	77.1	<0.0001
Q4	304	62.6	2,429	81.3	<0.0001
Paternal income					
Q1	556	49.3	2,453	62.6	<0.0001
Q2	410	54.0	2,260	72.3	<0.0001
Q3	549	61.8	3,404	77.6	<0.0001
Q4	572	66.2	5,056	84.5	<0.0001
Maternal occupational status					
No occupation	438	47.8	1,722	58.3	<0.0001
Occupation	1,777	60.0	12,005	77.9	<0.0001
Paternal occupational status					
No occupation	363	45.4	1,543	58.7	<0.0001
Occupation	1,736	60.5	11,703	78.5	<0.0001

Abbreviations: SES, socioeconomic status; Q, quartile. ¶Performed with chi-square.
1 EUR ≈ 9.83 SEK December 31st 2017. Q1: <18,652 EUR; Q2: 18,652 to 25,025 EUR; Q3: 25,026 to 32,309 EUR; Q4: >32,309 EUR.
