# Supplementary material for: Obesity in childhood, socioeconomic status, and completion of 12 or more school years: a prospective cohort study
Source: BMJ Open. 2021 Mar 11;11(3):e040432. doi: 10.1136/bmjopen-2020-040432 (PMC7957136; doi:10.1136/bmjopen-2020-040432)
Supplement: Supplementary data [file bmjopen-2020-040432supp003.pdf]

S3 Table. Adjusted odds ratio (99% CI); p-value of subjects completing ≥12 years of schooling stratified by group

	Childhood obesity cohort	Comparison group
Parental SES (ref=low)[a]		
Medium-low	1.64 (1.30 to 2.07); <0.0001	1.81 (1.59 to 2.07); <0.0001
Medium-high	2.43 (1.88 to 3.12); <0.0001	3.25 (2.82 to 3.75); <0.0001
High	3.22 (2.24 to 4.64); <0.0001	5.80 (4.81 to 6.99); <0.0001
Maternal education (ref=compulsory school)[b]		
Upper secondary school	1.64 (1.33 to 2.01); <0.0001	1.75 (1.56 to 1.96); <0.0001
University degree	2.33 (1.76 to 3.09); <0.0001	2.54 (2.20 to 2.93); <0.0001
Paternal education (ref=compulsory school)[b]		
Upper secondary school	1.29 (1.05 to 1.58); 0.0012	1.59 (1.42 to 1.79); <0.0001
University degree	1.46 (1.06 to 2.01); 0.0025	2.28 (1.94 to 2.67); <0.0001
Maternal income (ref=Q1)[c]		
Q2	1.28 (1.01 to 1.61); 0.0064	1.26 (1.11 to 1.42); <0.0001
Q3	1.26 (0.98 to 1.62); 0.019	1.30 (1.13 to 1.49); <0.0001
Q4	1.42 (1.04 to 1.95); 0.004	1.47 (1.25 to 1.73); <0.0001
Paternal income (ref=Q1)[c]		
Q2	1.19 (0.92 to 1.54); 0.075	1.44 (1.25 to 1.66); <0.0001
Q3	1.57 (1.22 to 2.02); <0.0001	1.79 (1.56 to 2.05); <0.0001
Q4	1.96 (1.51 to 2.55); <0.0001	2.63 (2.29 to 3.03); <0.0001
Maternal occupational status (ref=no occupation)[d]		
Occupation	1.42 (1.14 to 1.77); <0.0001	1.83 (1.61 to 2.08); <0.0001
Paternal occupational status (ref=no occupation)[d]		
Occupation	1.63 (1.30 to 2.04); <0.0001	1.90 (1.67 to 2.16); <0.0001

[a]Adjusted for migration background, ADHD/ADD, anxiety/depression, parental SES

[b]Adjusted for migration background, ADHD/ADD, anxiety/depression, maternal-, and paternal education

[c]Adjusted for migration background, ADHD/ADD, anxiety/depression, maternal-, and paternal income

[d]Adjusted for migration background, ADHD/ADD, anxiety/depression, maternal-, and paternal occupational status

Abbreviations: SES, socioeconomic status; Q, quartile.
Q1: <18,652 EUR; Q2: 18,652 to 25,025 EUR; Q3: 25,026 to 32,309 EUR; Q4: >32,309 EUR. 1 EUR ≈ 9.83 SEK December 31st 2017.
