# Supplementary material for: Obesity in childhood, socioeconomic status, and completion of 12 or more school years: a prospective cohort study
Source: BMJ Open. 2021 Mar 11;11(3):e040432. doi: 10.1136/bmjopen-2020-040432 (PMC7957136; doi:10.1136/bmjopen-2020-040432)
Supplement: Supplementary data [file bmjopen-2020-040432supp004.pdf]

S4 Table. Proportion of individuals who have undergone obesity treatment in childhood who completed ≥12 years of schooling by calendar year and by age at start of obesity treatment (n=3,942).

Individuals completing ≥12 years of schooling	n	%
Calendar year at start of obesity treatment		
1995-2001	229	62.6
2002-2008	1,262	58.1
2009-2014	745	53.1
Age at start of obesity treatment		
10 - 12.99	960	59.0
13 - 16.99	1,276	55.1
