# Supplementary material for: Obesity in childhood, socioeconomic status, and completion of 12 or more school years: a prospective cohort study
Source: BMJ Open. 2021 Mar 11;11(3):e040432. doi: 10.1136/bmjopen-2020-040432 (PMC7957136; doi:10.1136/bmjopen-2020-040432)
Supplement: Author's manuscript [file bmjopen-2020-040432.draft_revisions.pdf]

BMJ Open is committed to open peer review. As part of this commitment we make the peer review history of every article we publish publicly available.

When an article is published we post the peer reviewers' comments and the authors' responses online. We also post the versions of the paper that were used during peer review. These are the versions that the peer review comments apply to.

The versions of the paper that follow are the versions that were submitted during the peer review process. They are not the versions of record or the final published versions. They should not be cited or distributed as the published version of this manuscript.

BMJ Open is an open access journal and the full, final, typeset and author-corrected version of record of the manuscript is available on our site with no access controls, subscription charges or pay-per-view fees (http://bmjopen.bmj.com).

If you have any questions on BMJ Open's open peer review process please email info.bmjopen@bmj.com

Childhood obesity is negatively associated with completed educational level independent of socioeconomic status: a prospective cohort study

Journal:	*BMJ Open*
Manuscript ID	bmjopen-2020-040432
Article Type:	Original research
Date Submitted by the Author:	13-May-2020
Complete List of Authors:	Lindberg, Louise; Karolinska Institutet, Dept. Clinical Science, Intervention and Technology<br>Persson, Martina; Karolinska Institute, Department of Medicine, Clinical Epidemiology, Karolinska Institutet;<br>Liljeqvist, Pernilla; Karolinska Institutet, Dept. Clinical Science, Intervention and Technology<br>Hagman, Emilia; Karolinska Institutet, Dept. Clinical Science, Intervention and Technology<br>Marcus, Claude; Karolinska Institutet, Dept. Clinical Science, Intervention and Technology
Keywords:	Paediatric endocrinology < DIABETES & ENDOCRINOLOGY, EPIDEMIOLOGY, Paediatric endocrinology < PAEDIATRICS, PUBLIC HEALTH

SCHOLARONE™
Manuscripts

I, the Submitting Author has the right to grant and does grant on behalf of all authors of the Work (as defined in the below author licence), an exclusive licence and/or a non-exclusive licence for contributions from authors who are: i) UK Crown employees; ii) where BMJ has agreed a CC-BY licence shall apply, and/or iii) in accordance with the terms applicable for US Federal Government officers or employees acting as part of their official duties; on a worldwide, perpetual, irrevocable, royalty-free basis to BMJ Publishing Group Ltd ("BMJ") its licensees and where the relevant Journal is co-owned by BMJ to the co-owners of the Journal, to publish the Work in this journal and any other BMJ products and to exploit all rights, as set out in our licence.

The Submitting Author accepts and understands that any supply made under these terms is made by BMJ to the Submitting Author unless you are acting as an employee on behalf of your employer or a postgraduate student of an affiliated institution which is paying any applicable article publishing charge ("APC") for Open Access articles. Where the Submitting Author wishes to make the Work available on an Open Access basis (and intends to pay the relevant APC), the terms of reuse of such Open Access shall be governed by a Creative Commons licence – details of these licences and which Creative Commons licence will apply to this Work are set out in our licence referred to above.

Other than as permitted in any relevant BMJ Author's Self Archiving Policies, I confirm this Work has not been accepted for publication elsewhere, is not being considered for publication elsewhere and does not duplicate material already published. I confirm all authors consent to publication of this Work and authorise the granting of this licence.

For peer review only

Childhood obesity is negatively associated with completed educational level independent of socioeconomic status: a prospective cohort study

Louise Lindberg[1], Martina Persson[2,3,4], Pernilla Danielsson[1], Emilia Hagman[1*], Claude Marcus[1]

[1]Department of Clinical Science, Intervention and Technology, Division of Pediatrics, Karolinska Institutet, Stockholm, Sweden

[2]Department of Medicine Solna, Clinical Epidemiology Unit, Karolinska Institutet, Stockholm, Sweden

[3]Department of Diabetes and Endocrinology, Sachsska Children's Hospital Södersjukhuset, Stockholm, Sweden

[4]Department of Clinical Science and Education, Södersjukhuset, Karolinska Institutet, Stockholm, Sweden

*Corresponding author e-mail: emilia.hagman @ki.se (EH)

Other contributing authors e-mail: louise.lindberg@ki.se; martina.persson@ki.se; pernilla.danielsson.liljeqvist@ki.se; claude.marcus@ki.se

Keywords: Pediatric obesity, schools, epidemiology, social class, cohort study

Word count: 3574

For peer review only - http://bmjopen.bmj.com/site/about/guidelines.xhtml

Abstract

Objectives

Children with obesity achieve lower educational level compared with normal-weight peers. Parental socioeconomic status (SES) impacts both a child's academic achievement and risk of obesity. The degree to which the association between obesity and education depend on parental SES is unclear. Therefore, the primary aim is to investigate if individuals with obesity in childhood are less likely to complete ≥12 years of schooling, independently of parental SES. The secondary aim is to study how weight loss, level of education, and parental SES are associated.

Methods

In this nationwide prospective cohort study, children aged 10-17 years, recorded in the Swedish Childhood Obesity Treatment Register (BORIS), and aged 20 years or older at follow-up were included (n=3,942). A comparison group was matched by sex, year of birth, and living area (n=18,728). Parental SES was based on maternal and paternal level of education, income, and occupational status. The main outcome, completion of ≥12 years of schooling, was analysed with conditional logistic regression, and adjusted for group, migration background, ADHD/ADD, anxiety/depression, and parental SES.

Results

Among those with obesity in childhood, 56.7% completed ≥12 years in school compared with 74.4% in the comparison group ($p<0.0001$). High parental SES compared to low SES

For peer review only - http://bmjopen.bmj.com/site/about/guidelines.xhtml

was strongly associated with attained level of education in both children with and without obesity, adjusted odds ratio ([a]OR) [99% confidence interval (CI)]=5.40 [4.45 to 6.55]. However, obesity in childhood remains a strong risk factor of completing ≥12 school years, independently of parental SES, [a]OR=0.57 [0.51 to 0.63]. Successful obesity treatment increased the odds of completing ≥12 years in school even when taking parental SES into account, [a]OR=1.34 [1.04 to 1.72].

Conclusions

Individuals with obesity in childhood have lower odds of completing ≥12 years of schooling, independently of parental SES. Optimized obesity treatment may improve school results in this group.

Article summary / Strengths and limitation of this study

- In this prospective cohort study, we have been able to investigate the level of education among a large number of individuals who have obesity in childhood (n= 3,942) in comparison with a matched group (n=18,728).
- The study design of using longitudinal data from several national registers provided the opportunity to control for important confounding factors, such as neuropsychiatric disorders, anxiety, depression and family socioeconomic status.
- Factors such as free education, school lunches, and students' health care may have an impact on the generalizability of our data to other populations.

For peer review only - http://bmjopen.bmj.com/site/about/guidelines.xhtml

Introduction

During the last 40 years, the prevalence of childhood obesity has increased exponentially in many parts of the world.[1] Childhood obesity is associated with increased risks of somatic morbidity and risk of premature death in adulthood.[2-5] Psychosocial maladjustment, anxiety and depression are also prevalent[6 7] and may contribute to the obesity-related long-term morbidity and mortality.[5] Most studies report that children with obesity more often have lower school grades and reach a lower level of education compared with normal-weight peers,[8-10] but in a recent study, only girls with obesity were affected.[11] We have previously confirmed the association between obesity and lower attained level of education among both girls and boys.[9] We also found that successful obesity treatment was positively correlated with completing ≥12 school years, although without taking socioeconomic status (SES) into account.[9]

Low parental SES is a well-established risk factor for both childhood obesity and poorer academic achievement.[12] In particular, parental education has been demonstrated to influence the child's performance at school.[13-15] In addition, depression[16] and neuropsychiatric disorders, including Attention Deficit Disorder with or without Hyperactivity (ADHD/ADD),[17] may adversely affect school results. Depression and neuropsychiatric disorders are more common in children with obesity compared to the general population.[9 18]. Investigating the impact of obesity alone on attained level of education requires that these and other confounders are considered. The primary aim of this study was to disentangle the association of childhood obesity and parental SES on completed level of education. The

For peer review only - http://bmjopen.bmj.com/site/about/guidelines.xhtml

secondary aim was to study if positive effects of weight loss on attained level of education is affected by parental SES.

Methods

Study population

This prospective cohort study included children with obesity,[19] aged 10-17 years at the start of obesity treatment (December 1994 to December 2015), and aged 20 years or older and living in Sweden at follow-up (December 31st 2017; n=3,942). Data on subjects receiving treatment for childhood obesity was collected from the Swedish Childhood Obesity Treatment Register (BORIS). BORIS has been thoroughly described elsewhere,[20] but in short: The main purpose of the register is quality assessment and long-term evaluation of childhood obesity treatment. Treatment is individualized and consists primarily of lifestyle modification (i.e. diet and physical activity).

A comparison group from the general population was randomly identified using the Swedish Total Population Register and matched by sex, year of birth, and living area at the year obesity treatment was initiated (n=18,728). Using density matching without replacement, five individuals were matched to each individual from the childhood obesity cohort. Siblings of children registered in the childhood obesity cohort were excluded from the comparison group. Children with a diagnosis of mental retardation or genetic syndromes were excluded from both the childhood obesity cohort and the comparison group (Fig 1 and S1 Table).

Fig 1. Participant flowchart.

For peer review only - http://bmjopen.bmj.com/site/about/guidelines.xhtml

Families were informed in written or verbal about data collection in the Swedish Childhood Obesity Treatment Register. Post an opt-out approval by parents/guardians, data of the children's weight and height were recorded by the local health care provider during treatment visits. There was no data of weight and height of individuals in the comparison group. However, less than 1% of the individuals in the comparison group were found in the National Patient Register with a diagnosis of obesity in childhood. In Sweden, health care is free of charge for children and adolescents until 18 years of age. The study was approved by the regional Ethics Committee in Stockholm, Sweden (No. 2016/922-31/1).

Data sources

Using the Swedish identity number, which is unique to each resident in Sweden, data from several national registers were linked [21]. Data on education, income, and occupational status were obtained from the Longitudinal Integration Database for Health Insurance and Labour Market Studies. Information on migration background for children and their parents was obtained from the Swedish Total Population Register [22]. Both these registers are held by Statistics Sweden, a governmental agency that collects and provide official statistics (www.scb.se/en).

Data on diagnoses of mental retardation, genetic syndromes, ADHD/ADD, anxiety, and depression were identified based on codes according the International Classification of Diseases (10th revision; S1 Table) and retrieved from the National Patient Register [23]. Information on prescriptions of psychostimulant drugs for ADHD/ADD as well as antidepressants and tranquilizers for anxiety and depression were identified using the Anatomical Therapeutic Chemical classification system (S1 Table) retrieved from the

For peer review only

For peer review only - http://bmjopen.bmj.com/site/about/guidelines.xhtml

Swedish Prescribed Drug Register [24]. Data on deaths were retrieved from the Cause of Death Register [25]. These registers are held and were linked by the governmental agency the National Board of Health and Welfare (www.socialstyrelsen.se/english).

Definition of outcome

The main outcome was defined as completion of ≥12 years of schooling, and based on the International Standard Classification of Education [26]. In Sweden, children start school at the age of 6 or 7 years and attend compulsory school for 9 years. Upper secondary school includes three additional years of schooling and provides the requirements to attend higher education. Usually students graduate from secondary school at 18 or 19 years of age. All education in Sweden is free of charge. Students in compulsory and upper secondary school are provided school lunches and health care at schools, also free of charge.

Definition of main exposures

Body Mass Index Standard Deviation Score (BMI SDS) is standardized by sex and age and used to measure degree of obesity in growing children [19]. Baseline measures (continuous) were used for the variable BMI SDS at start of treatment. Response to obesity treatment was based on the change of BMI SDS from the first to the last clinical visit and categorized into four groups [27 28]: good response, a reduction of BMI SDS by 0.25 units or more; no response, a change of BMI SDS by +/- 0.25 units; poor response, an increase of BMI SDS by 0.25 units or more; and dropouts, children with less than one year between their first and last measure or without clinical follow-up after their first registered visit.

For peer review only

For peer review only - http://bmjopen.bmj.com/site/about/guidelines.xhtml

Parental SES was based on maternal and paternal level of education, income, and occupational status at the year the child turned 15 years, which is about the same time as the child starts upper secondary school. In case of the child was adopted, the SES of adoptive parents was used (childhood obesity cohort n=24 and comparison group n=164). The rationale of treating SES as a composite variable was to capture more of the social context and a potential inequality embedded there. Thus, by taking three variables into account instead of one, we get a more wide and robust measure of SES. Maternal and paternal educational level was categorized into compulsory school, upper secondary school, or university degree. Annual disposable income was used to reflect maternal and paternal economic capacity. The annual disposable income includes all taxable (direct labour income, capital gains from shares etc.) and tax-free income (housing and child benefits, student aid etc.), minus final tax and other negative transfers such as capital loss from shares and properties. Disposable income from different years was converted to 2017 prices using the Consumer Price Index for Sweden provided by Statistics Sweden (www.scb.se/en). Income was categorized into quartiles based on data from the parents in the comparison group**.** No occupation was defined as either unemployment 6 months or more, or income from long-term sick leave exceeding any income from the individual's gross salary. Individuals considered to have an occupation included those registered as employed or having an income from student grants/loan equivalent to full-time studies for at least one semester. Parental SES was weighted according to maternal and paternal education (0, 1, 2), income (0, 1, 2, 3), and occupational status (0, 1). The mean parental SES score (i.e. the sum of the mother's and father's all SES indicators divided by two) was applied to their child. The SES-variable was

For peer review only - http://bmjopen.bmj.com/site/about/guidelines.xhtml

categorized into four levels; low SES (0 to 1.5 points), medium-low SES (2 to 3 points), medium-high SES (3.5 to 4.5 points), and high SES (5 to 6 points).

The prevalence of both ADHD/ADD [9] and depression [18] is higher in children with obesity and may negatively influence attained level of education [9 16 17]. ADHD/ADD and anxiety/depression in children were identified based on diagnosis or dispensed prescribed medication (S1 Table).

Definition of covariates

The migration background of children in Sweden may impact school achievements and is therefore an important factor to control for in the analyses [9 14]. Migration background was categorized as Nordic or non-Nordic. Nordic was defined as born in a Nordic country (Sweden, Norway, Denmark, Finland, or Iceland) with one or two parents born in the Nordic. Children were classified as non-Nordic if born outside the Nordic or born in the Nordic with two parents born outside the Nordic. Other covariates included sex and age (continuous) at start of obesity treatment.

Statistical analysis

Descriptive statistics are presented as means and confidence intervals (CI), medians and interquartile ranges (IQR), or frequencies and percentages. Conditional logistic regression was used to calculate odds ratios (OR's) with 99% CI for the main outcome, i.e. completing ≥12 school years. Conditional logistic regression was used above ordinary logistic regression as the childhood obesity cohort and the comparison group were matched by several variables. Independent variables included in the adjusted analyses were migration background,

For peer review only - http://bmjopen.bmj.com/site/about/guidelines.xhtml

ADHD/ADD, anxiety/depression, parental SES, and group (childhood obesity cohort or comparison group) in analyses including both groups, otherwise stratified by group. Interaction between childhood obesity and parental SES was tested. The association of each SES indicator on the odds of completing ≥12 school years was also analysed separately for mothers and fathers. Sensitivity analyses excluding individuals with ADHD/ADD or anxiety/depression were performed.

Secondary analyses were performed within the childhood obesity cohort to examine associations between patient characteristics and completed educational level. In ordinary logistic regressions, odds were adjusted for sex, migration background, parental SES, age and BMI SDS at start of treatment, treatment response, ADHD/ADD, and anxiety/depression. We tested for possible interaction between parental SES and treatment response for the odds of completing ≥12 years of schooling.

As missing data on parental SES was rare in both groups (Table 1), records with missing data were excluded from the analyses, i.e. data were not imputed. P-values <0.01 were considered statistically significant. All analyses were performed using SAS statistical software (version 9.4, Cary, NC, USA).

For peer review only

For peer review only - http://bmjopen.bmj.com/site/about/guidelines.xhtml

212

Table 1. Characteristics of the participants (n=22,670)

	Childhood obesity cohort (n=3,942)		Comparison group (n=18,728)	
	n	%	n	%
Female sex	1,825	46.3	8,701	46.5
Nordic	2,905	73.7	14,048	75.0
Age at end of follow-up (years)	23.4*	21.4-26.3*	23.4*	21.5-26.3*
ADHD/ADD	617	15.7	1,044	5.6
Anxiety/Depression	831	21.1	2,158	11.5
Parental SES				
Low	867	22.0	2,700	14.4
Medium-low	1,540	39.1	6,023	32.1
Medium-high	1,159	29.4	6,587	35.2
High	355	9.0	3,218	17.2
Missing	21	0.5	200	1.1
Maternal education				
Compulsory school	1,582	40.1	5,615	30.0
Upper secondary school	1,541	39.1	7,167	38.3
University degree	730	18.5	5,383	28.7
Missing	89	2.3	563	3.0
Paternal education				
Compulsory school	1,273	32.3	4,402	23.5
Upper secondary school	1,890	48.0	9,095	48.6
University degree	482	12.2	3,912	20.9
Missing	297	7.5	1,319	7.0
Maternal income				
Q1	1,173	29.8	4,887	26.1
Q2	1,281	32.5	5,843	31.2
Q3	931	23.6	4,585	24.5
Q4	486	12.3	2,988	15.9
Missing	71	1.8	425	2.3
Paternal income				
Q1	1,127	28.6	3,917	20.9
Q2	760	19.3	3,128	16.7
Q3	889	22.5	4,389	23.4
Q4	864	21.9	5,986	32.0
Missing	302	7.7	1,308	7.0
Maternal occupational status				
Occupation	2,963	75.2	15,405	82.2
No occupation	916	23.2	2,956	15.8
Missing	63	1.6	367	2.0
Paternal occupational status				
Occupation	2,868	72.8	14,906	79.6
No occupation	800	20.3	2,627	14.0
Missing	274	6.9	1,195	6.4

Data are n % if not else stated. Abbreviation: SES, socioeconomic status; Q, quartile.
*Median with interquartile ranges

213

214

For peer review only - http://bmjopen.bmj.com/site/about/guidelines.xhtml

Results

In total, 3,942 individuals in the childhood obesity cohort and 18,728 individuals in the comparison group were included in the study (Table 1). In both groups, 46% of the participants were girls and the median age at follow-up was 23.4 years. The proportions of individuals of Nordic origin were similar in both groups (73.7% vs. 75.0%; p=0.02). Despite the groups being matched for living area, a greater proportion of children with obesity grew up in households with low SES compared with the comparison group (22.0% vs. 14.4%; p<0.0001).

Parental SES, childhood obesity and the child's attained level of education

In the childhood obesity cohort, 56.7% completed ≥12 years in school, compared with 74.4% in the comparison group. Girls more frequently completed ≥12 years of schooling than boys in both groups (S2 Table).

Higher parental SES was positively associated with completion of ≥12 years of schooling in both the childhood obesity cohort and in the comparison group (Fig 2). The odds were more than five times higher among children growing up in high SES households compared with those growing up in low SES households (Table 2). However, even when taking parental SES and other risk factors into account, individuals in the childhood obesity cohort were almost half as likely to complete ≥12 school years compared with those in the comparison group (Table 2). The adjusted odds ratio (^{a}OR) [99% CI] to complete ≥12 years of schooling

For peer review only - http://bmjopen.bmj.com/site/about/guidelines.xhtml

for the childhood obesity cohort versus the comparison group were lower in the higher level of SES: [a]OR low parental SES = 0.69 [0.50 to 0.95], p=0.0026; [a]OR medium-low parental SES = 0.59 [0.48 to 0.72], p<0.0001; [a]OR medium-high parental SES = 0.46 [0.35 to 0.60], p<0.0001; [a]OR high parental SES = 0.27 [0.14 to 0.54], p<0.0001. P-value for interaction test for childhood obesity and parental SES reaches a p of 0.0015.

The association of parental SES on school performance was more pronounced in the comparison group than in the childhood obesity cohort. For example, stratified analyses show that adjusted OR of completing ≥12 years in school in low SES compared with high SES in the comparison group was 0.17 (99% CI 0.14 to 0.21, p<0.0001) and in the childhood obesity cohort 0.31 (99% CI 0.22 to 0.45, p<0.0001). Regardless of how we divide the childhood obesity population (into age- or calendar year at start of obesity treatment, SES sub-scores), large differences between the two groups remain (Table 2 and S3 Table).

Fig 2. Percentage of individuals completing ≥12 years of schooling by SES level in the childhood obesity cohort (n=3,921, solid bars) and the comparison group (n=18,528, dotted bars).

For peer review only - http://bmjopen.bmj.com/site/about/guidelines.xhtml

Table 2. Crude and adjusted odds ratio (99% CI); p-value of subjects completing ≥12 years of schooling

	Crude estimates	Model 1, n=22 449	Model 2, n=20 610	Model 3, n=20 691	Model 4, n=20 885	Model 5, n=20 420
Childhood obesity cohort vs. comparison group	0.44 (0.40 to 0.48); <0.0001	0.57 (0.51 to 0.63); <0.0001	0.55 (0.50 to 0.62); <0.0001	0.54 (0.48 to 0.61); <0.0001	0.53 (0.48 to 0.59); <0.0001	0.59 (0.52 to 0.66); <0.0001
Non-Nordic vs. Nordic	0.70 (0.63 to 0.78); <0.0001	0.85 (0.75 to 0.97); 0.001	0.77 (0.67 to 0.88); <0.0001	0.87 (0.76 to 0.99); 0.0068	0.81 (0.71 to 0.92); <0.0001	0.97 (0.84 to 1.12); 0.5687
ADHD/ADD vs. non-ADHD/ADD	0.19 (0.17 to 0.23); <0.0001	0.28 (0.24 to 0.33); <0.0001	0.27 (0.22 to 0.33); <0.0001	0.27 (0.22 to 0.32); <0.0001	0.26 (0.22 to 0.31); <0.0001	0.28 (0.23 to 0.34); <0.0001
Anxiety/depression vs. no anxiety/depression	0.31 (0.27 to 0.35); <0.0001	0.39 (0.34 to 0.45); <0.0001	0.36 (0.31 to 0.41); <0.0001	0.38 (0.33 to 0.44); <0.0001	0.39 (0.33 to 0.44); <0.0001	0.36 (0.31 to 0.42); <0.0001
Parental SES						
Medium-low vs. low parental SES	1.74 (1.54 to 1.97); <0.0001	1.69 (1.48 to 1.93); <0.0001				
Medium-high vs. low parental SES	3.33 (2.92 to 3.81); <0.0001	2.99 (2.59 to 3.45); <0.0001				
High vs. low parental SES	6.21 (5.19 to 7.44); <0.0001	5.40 (4.45 to 6.55); <0.0001				
Maternal education						
Upper secondary school vs. compulsory school	1.88 (1.69 to 2.08); <0.0001		1.63 (1.45 to 1.83); <0.0001			1.51 (1.34 to 1.71); <0.0001
University degree vs. compulsory school	3.23 (2.86 to 3.66); <0.0001		2.45 (2.12 to 2.83); <0.0001			2.17 (1.87 to 2.53); <0.0001
Paternal education						
Upper secondary school vs. compulsory school	1.88 (1.69 to 2.09); <0.0001		1.58 (1.40 to 1.77); <0.0001			1.42 (1.26 to 1.60); <0.0001
University degree vs. compulsory school	3.07 (2.66 to 3.55); <0.0001		2.09 (1.77 to 2.45); <0.0001			1.81 (1.53 to 2.15); <0.0001
Maternal income						
Q2 vs. Q1	1.28 (1.15 to 1.43); <0.0001			1.25 (1.10 to 1.41); <0.0001		1.07 (0.93 to 1.23); 0.2065
Q3 vs. Q1	1.45 (1.28 to 1.63); <0.0001			1.35 (1.17 to 1.55); <0.0001		1.05 (0.90 to 1.23); 0.4224
Q4 vs. Q1	1.86 (1.60 to 2.15); <0.0001			1.52 (1.28 to 1.81); <0.0001		1.07 (0.88 to 1.28); 0.3831
Paternal income						
Q2 vs. Q1	1.36 (1.19 to 1.55); <0.0001			1.31 (1.13 to 1.51); <0.0001		1.07 (0.91 to 1.26); 0.2740
Q3 vs. Q1	1.90 (1.67 to 2.17); <0.0001			1.70 (1.48 to 1.96); <0.0001		1.24 (1.05 to 1.46); 0.0009
Q4 vs. Q1	2.95 (2.58 to 3.36); <0.0001			2.47 (2.13 to 2.86); <0.0001		1.58 (1.33 to 1.88); <0.0001
Maternal occupational status						
Occupation vs. no occupation	2.15 (1.92 to 2.40); <0.0001				1.72 (1.51 to 1.96); <0.0001	1.38 (1.19 to 1.61); <0.0001
Paternal occupational status						
Occupation vs. no occupation	2.24 (1.99 to 2.52); <0.0001				1.79 (1.57 to 2.04); <0.0001	1.41 (1.20 to 1.65); <0.0001

Abbreviations: CI, confidence interval; SES, socioeconomic status.
Model 1: Variables included were group (childhood obesity cohort vs. comparison group), migration background, ADHD/ADD, anxiety/depression, and parental SES
Model 2: Variables included were group, migration background, ADHD/ADD, anxiety/depression, maternal-, and paternal education
Model 3: Variables included were group, migration background, ADHD/ADD, anxiety/depression, maternal-, and paternal income
Model 4: Variables included were group, migration background, ADHD/ADD, anxiety/depression, maternal-, and paternal occupational status.
Model 5: Variables included were group, migration background, ADHD/ADD, anxiety/depression, maternal-, and paternal education, income, and occupational status.

For peer review only - http://bmjopen.bmj.com/site/about/guidelines.xhtml

Of note, having a non-Nordic-, compared to a Nordic background, was associated with reduced odds to complete 12 or more years of schooling (Table 2). This was however not observed in boys in the childhood obesity cohort (p=0.68). Further, excluding individuals with ADHD/ADD or anxiety/depression in sensitivity analyses, did not alter the association between childhood obesity and attained level of education.

Degree of obesity and treatment response on completed educational level in children with obesity

In the childhood obesity cohort, the mean age at start of treatment was 13.5 years (99% CI: 13.45 to 13.62) and the mean BMI SDS was +2.91 (99% CI: 2.89 to 2.92). The median treatment duration was 2.83 years (IQR: 1.86 to 4.42) and the mean treatment response, calculated using BMI SDS from the first to the last clinical visit, was -0.13 (99% CI: -0.15 to -0.10, n=2,709, 68.7%, dropouts excluded). At baseline, 50.7% had obesity and 49.3% had morbid obesity. At last measured weight and height, 33.4% had obesity and 47.2% had morbid obesity, while 19.4% of the children no longer had obesity (dropouts excluded).

A greater degree of obesity at start of treatment lowered the odds of completing ≥12 years of schooling, [a]OR [99% CI] = 0.51 [0.40 to 0.64], p<0.0001, per one unit increase in BMI SDS, while age at start of obesity treatment did not influence the outcome (Table 3). Treatment response was categorized as good response (n=847), no response (n=1 315), poor response (n=547), and dropouts (n=1,233). Of those with good treatment response, 67% completed ≥12 years in school compared with 58%, 52%, and 50% in the groups with no or poor response, and dropouts, respectively (p<0.0001). Within all SES groups, except for high SES, greater treatment response resulted in higher odds of completing ≥12 years in school (Fig 3).

For peer review only - http://bmjopen.bmj.com/site/about/guidelines.xhtml

Dropouts were less likely to complete ≥12 years in school compared with non-responders (Table 3) but this was not observed in the high SES group (Fig 3). Duration of treatment differed between children with good and poor treatment response (3.5 vs. 4.0 years; $p<0.0001$). In the childhood obesity cohort, the association between parental SES and odds of completing ≥12 years of schooling was not modified by treatment response ($p=0.603$).

For peer review only

For peer review only - http://bmjopen.bmj.com/site/about/guidelines.xhtml

Table 3. Crude and adjusted odds ratios of completing ≥12 years of schooling in the childhood obesity cohort

	Odds ratio (99% CI) performed with ordinary logistic regression; p value		
	Crude estimates	Model 1, n=3 921	Model 2, n=3 575
Sex (girls vs. boys)	1.26 (1.06 to 1.48); 0.0004	1.30 (1.08 to 1.56); 0.0002	1.29 (1.06 to 1.56); 0.0008
Migration background (non-Nordic vs. Nordic)	0.78 (0.65 to 0.94); 0.0006	0.74 (0.60 to 0.91); 0.0002	0.69 (0.55 to 0.87); <0.0001
ADHD/ADD (yes vs. no)	0.31 (0.25 to 0.40); <0.0001	0.35 (0.27 to 0.45); <0.0001	0.33 (0.26 to 0.44); <0.0001
Anxiety/depression (yes vs. no)	0.43 (0.35 to 0.53); <0.0001	0.45 (0.36 to 0.57); <0.0001	0.43 (0.34 to 0.55); <0.0001
BMI SDS at start of treatment	0.47 (0.38 to 0.58); <0.0001	0.51 (0.40 to 0.64); <0.0001	0.48 (0.38 to 0.62); <0.0001
Age at start of treatment	0.96 (0.93 to 1.01); 0.0234	1.01 (0.96 to 1.06); 0.60	1.01 (0.96 to 1.06); 0.805
Treatment response			
Good response vs. no response	1.46 (1.15 to 1.85); <0.0001	1.34 (1.04 to 1.72); 0.0031	1.28 (0.98 to 1.67); 0.0182
Poor response vs. no response	0.78 (0.60 to 1.02); 0.0167	0.72 (0.54 to 0.96); 0.0028	0.71 (0.53 to 0.96); 0.003
Dropouts vs. no response	0.71 (0.58 to 0.87); <0.0001	0.69 (0.55 to 0.86); <0.0001	0.68 (0.54 to 0.87); <0.0001
Parental SES			
Medium-low vs. low parental SES	1.63 (1.31 to 2.03); <0.0001	1.56 (1.24 to 1.98); <0.0001	
Medium-high vs. low parental SES	2.40 (1.90 to 3.05); <0.0001	2.19 (1.69 to 2.83); <0.0001	
High vs. low parental SES	2.99 (2.11 to 4.22); <0.0001	2.83 (1.95 to 4.12); <0.0001	
Maternal education			
Upper secondary school vs. compulsory school	1.74 (1.44 to 2.09); <0.0001		1.57 (1.27 to 1.94); <0.0001
University degree vs. compulsory school	2.47 (1.94 to 3.16); <0.0001		2.23 (1.67 to 2.98); <0.0001
Paternal education			
Upper secondary school vs. compulsory school	1.48 (1.23 to 1.79); <0.0001		1.26 (1.03 to 1.55); 0.004
University degree vs. compulsory school	1.92 (1.44 to 2.56); <0.0001		1.41 (1.01 to 1.95); 0.0076

Abbreviations: CI, confidence interval; BMI SDS, BMI standard deviation score; SES, socioeconomic status.
Model 1: Variables included were sex, migration background, ADHD/ADD, anxiety/depression, BMI SDS- and age at start of treatment, treatment response, and parental SES.
Model 2: Variables included were sex, migration background, ADHD/ADD, anxiety/depression, BMI SDS- and age at start of treatment, treatment response, maternal-, and paternal education.

280

For peer review only - http://bmjopen.bmj.com/site/about/guidelines.xhtml

Fig 3. Crude percentage of individuals with obesity in childhood (n=3,921) completing ≥12 school years by parental SES and treatment response (black bars=dropouts; white bars=poor response; striped bars=no response; dotted bars=good response). p=p for trend; n=refers to number of individuals in each category.

Discussion

For peer review only

In this prospective cohort study, we have compared level of education among individuals recorded in the Swedish Childhood Obesity Treatment Register, and a comparison group matched by sex, year of birth, and living area. Individuals in the childhood obesity cohort were half as likely to complete 12 or more years of schooling, independently of parental socioeconomic status (SES).

Among individuals from high SES families, those in the childhood obesity cohort were approximately one third as likely to complete ≥12 years of schooling as individuals from high SES families in the comparison group. Furthermore, our results indicate that parental SES was more important to complete ≥12 school years in the comparison group than in the childhood obesity cohort. An association between obesity and impaired academic achievements has been demonstrated before.[8,9] To which extent other psychosocial factors contribute to this finding has not been evaluated. It is well established that low parental SES,[13-15] immigrant background,[9,14] ADHD/ADD,[9,17] and depression,[9,14,16] may negatively affect performance in school. These factors are also overrepresented in the paediatric population with obesity. In this study, we can confirm that low parental SES, neuropsychiatric disorders, and anxiety/depression are more common in children with obesity and contribute to the decreased odds of completing ≥12 school years. However, we found that obesity in childhood is a considerable risk factor for not

For peer review only - http://bmjopen.bmj.com/site/about/guidelines.xhtml

completing ≥12 years of schooling even after taking these and other important risk factors into account.

Obesity treatment outcome and educational level

We identified a positive association between weight-loss during obesity treatment and completed educational level, which confirms our previously reported data unadjusted for SES [9]. Children with good treatment response, compared with those with no response, were more likely to complete ≥12 school years in all SES groups. A decrease of 0.25 BMI SDS, that has previously been shown to improve metabolic health [27], we can now also show may have a positive association on completing ≥12 years of schooling. However, the positive effects of successful obesity treatment did not compensate for the observed SES differences on attained level of education.

In Sweden, 12 years of schooling is not mandatory, but the long-term social effects are considerable for those who fail. It has been estimated that 50% of those who fail to complete ≥12 school years face a situation of being left outside the society with poor psychosocial health and large costs for the society [29]. Thus, it is possible that school failure will further worsen the health perspective of adolescents with obesity.

Potential mechanisms between obesity, weight loss, and school performance

The mechanisms by which obesity influences school performance are unclear but are likely multifactorial and complex. Possible mechanisms may include psychosocial aspects such as stigma[30] and increased risk for anxiety and depression.[7] Physiological mechanisms may be mediated by anatomical changes such as atrophy of the frontal lobe and reduced hippocampal volume have been demonstrated in individuals with obesity.[31] Some of the reported cognitive

For peer review only - http://bmjopen.bmj.com/site/about/guidelines.xhtml

dysfunctions in children and adolescents with obesity include slower response times when performing visuospatial attention tasks,[32] diminished executive functions, such as working memory,[33] and slower cognitive performance speed[34]. In addition, obesity-related comorbidities such as insulin resistance,[35] type 2 diabetes mellitus,[36] chronic low-grade inflammation,[37] and the metabolic syndrome[38] have also been associated with impaired executive function, memory performance, attention, and cognitive flexibility. A high fat diet in rodents demonstrated detrimental effects on memory and executive functions.[31]

At least some of these effects seem to be reversible, which may add to the positive associations we observed of weight loss on school outcome. Weight loss may have a direct positive effect on cognitive functions.[39] Extensive weight loss via bariatric surgery improves insulin sensitivity and decreases systematic inflammation,[40] and it has been suggested that these factors affect cognitive functions.[41]

However, children with obesity are often stigmatized,[42] have a low self-esteem, and are exposed to bullying and social exclusion.[43] All these factors have most likely a negative impact on school performance. In contrast, a strong social network is most probably an important factor both for good treatment response and achievement in school. An inverse association has been observed between familial social support and child weight status[44] Thus, it is likely that both negative social effects of obesity and obesity-related morbidity, as well as genetic factors, contribute to the adverse association of childhood obesity on completed educational level.

Limitations

Using longitudinal data from several national registers provided an opportunity to assess the impact of obesity on completed educational level, adjusted for several important confounders. Data on both exposure and outcome were prospectively collected and defined according to standardized international classifications.[19 26]

For peer review only - http://bmjopen.bmj.com/site/about/guidelines.xhtml

However, some important limitations should be recognized. We did not have anthropometric data on children in the comparison group. There is no representative national data on children with obesity in Sweden. As our comparison group includes children with obesity, although likely less than 1% according to obesity diagnoses found in the National Patient Register, odds of lower level of education associated with obesity might be underestimated. It is also important to consider that children receiving obesity treatment may not be representative of all children with obesity. However, such bias is reduced as a large proportion of children are referred to treatment from school. It should be noted that parental SES was based on data from one specific year in the child's life and not over the child's entire adolescent lifetime, and that the SES indicators used may not reflect the whole SES spectrum. In addition, the impact of anxiety and depression on educational level may be underestimated since these conditions often are under-diagnosed. Lastly, factors such as free education, school lunches, and students' health care may have an impact on the generalizability of our data to other populations.

Conclusion

In this longitudinal, population-based study, individuals with obesity in childhood were less likely to complete 12 or more years of schooling, compared with a group from the general population. The odds associated with obesity remained significantly increased even after taking parental SES and other important risk factors, such as ADHD, into consideration. The underlying mechanisms are unclear but previous studies indicate that the effects of obesity both involve psychosocial effects and cognitive functions. The negative impact of childhood obesity on educational level could partly be reversed by successful obesity treatment in childhood. Results from this study underline the wide effects of childhood obesity on public health and the importance of continued efforts to reduce the prevalence of obesity in children. It is of clinical

For peer review only

For peer review only - http://bmjopen.bmj.com/site/about/guidelines.xhtml

importance to increase awareness of the potential need for extra support at school of children and adolescents with obesity.

Acknowledgements

The authors would like to thank all local health care professionals in Sweden working with children and adolescents with obesity and their valuable work with BORIS, and the BORIS steering committee for establishing and maintaining the register.

For peer review only

Author contributions

Conceptualization: Louise Lindberg, Pernilla Danielsson, Martina Persson, Claude Marcus, Emilia Hagman.

Data curation: Louise Lindberg.

Formal analysis: Louise Lindberg, Emilia Hagman.

Funding acquisition: Louise Lindberg, Claude Marcus, Emilia Hagman.

Methodology: Louise Lindberg, Emilia Hagman.

Project administration: Louise Lindberg, Emilia Hagman.

Supervision: Martina Persson, Emilia Hagman, Pernilla Danielsson, Claude Marcus.

Writing – original draft: Louise Lindberg.

Writing – review & editing: Louise Lindberg, Pernilla Danielsson, Martina Persson, Claude Marcus, Emilia Hagman.

Funding statement

This study was supported by funds to LL by Crown Princess Lovisa's Foundation (2017-00348), Samariten Foundation (2017-0305), the Stockholm FreeMason Foundation for Children's Welfare, Sällskapet Barnavård, Anna-Lisa och Arne Gustafssons Foundation,

For peer review only - http://bmjopen.bmj.com/site/about/guidelines.xhtml

Solstickan Foundation and Sven Jerring Foundation. EH was supported by the Swedish Society for Medical Research, Fredrik and Ingrid Thuring's Foundation (2017-00309) and Magnus Bergvall Foundation (2017-02113), and CM by the Swedish Heart and Lung Foundation (20150790).

Data Sharing statement

The data that support the findings of this study contains sensitive information. Restrictions therefore apply to the availability of these data, which were used under license for the current study, and so are not publicly available. According to Swedish law and the General Data Protection Regulation, the authors are not permitted to share the datasets used in this study with third parties. Given that an ethical approval is obtained, any individual may apply for data from Statistics Sweden via information@scb.se, the Swedish National Board of Health and Welfare via registerservice@socialstyrelsen.se, and the Swedish Childhood Obesity Treatment Register via http://www.e-boris.se/kontaktuppgifter/.

Competing interests

All authors have completed the ICMJE uniform disclosure form at www.icmje.org/coi_disclosure.pdf and declare: no support from any organisation for the submitted work; no financial relationships with any organisations that might have an interest in the submitted work in the previous three years; no other relationships or activities that could appear to have influenced the submitted.

For peer review only - http://bmjopen.bmj.com/site/about/guidelines.xhtml

References

1. Abarca-Gómez L, Abdeen ZA, Hamid ZA, et al. Worldwide trends in body-mass index, underweight, overweight, and obesity from 1975 to 2016: a pooled analysis of 2416 population-based measurement studies in 128.9 million children, adolescents, and adults. *The Lancet* 2017 doi: 10.1016/S0140-6736(17)32129-3
2. Hagman E, Danielsson P, Brandt L, et al. Association between impaired fasting glycaemia in pediatric obesity and type 2 diabetes in young adulthood. *Nutr Diabetes* 2016;6(8):e227. doi: 10.1038/nutd.2016.34 [published Online First: 2016/08/23]
3. Hagman E, Danielsson P, Elimam A, et al. The effect of weight loss and weight gain on blood pressure in children and adolescents with obesity. *Int J Obes (Lond)* 2019;43(10):1988-94. doi: 10.1038/s41366-019-0384-2 [published Online First: 2019/06/04]
4. Lindberg L, Danielsson P, Persson M, et al. Association of childhood obesity with risk of early all-cause and cause-specific mortality: A Swedish prospective cohort study. *PLoS medicine* 2020;17(3):e1003078. doi: 10.1371/journal.pmed.1003078 [published Online First: 2020/03/19]
5. Reilly JJ, Kelly J. Long-term impact of overweight and obesity in childhood and adolescence on morbidity and premature mortality in adulthood: systematic review. *Int J Obes (Lond)* 2011;35(7):891-8. doi: 10.1038/ijo.2010.222
6. Viner RM, Cole TJ. Adult socioeconomic, educational, social, and psychological outcomes of childhood obesity: a national birth cohort study. *BMJ* 2005;330(7504):1354. doi: 10.1136/bmj.38453.422049.E0 [published Online First: 2005/05/20]
7. Lindberg L, Hagman E, Danielsson P, et al. Anxiety and depression in children and adolescents with obesity: a nationwide study in Sweden. *BMC medicine* 2020;18(1):30. doi: 10.1186/s12916-020-1498-z [published Online First: 2020/02/23]
8. Laitinen J, Power C, Ek E, et al. Unemployment and obesity among young adults in a northern Finland 1966 birth cohort. *International journal of obesity and related metabolic disorders : journal of the International Association for the Study of Obesity* 2002;26(10):1329-38. doi: 10.1038/sj.ijo.0802134
9. Hagman E, Danielsson P, Brandt L, et al. Childhood Obesity, Obesity Treatment Outcome, and Achieved Education: A Prospective Cohort Study. *The Journal of adolescent health : official publication of the Society for Adolescent Medicine* 2017 doi: 10.1016/j.jadohealth.2017.04.009 [published Online First: 2017/07/12]
10. Karnehed N, Rasmussen F, Hemmingsson T, et al. Obesity and attained education: cohort study of more than 700,000 Swedish men. *Obesity* 2006;14(8):1421-28.
11. French SA, Wall M, Corbeil T, et al. Obesity in Adolescence Predicts Lower Educational Attainment and Income in Adulthood: The Project EAT Longitudinal Study. *Obesity (Silver Spring)* 2018;26(9):1467-73. doi: 10.1002/oby.22273 [published Online First: 2018/09/19]
12. Shrewsbury V, Wardle J. Socioeconomic status and adiposity in childhood: a systematic review of cross-sectional studies 1990-2005. *Obesity (Silver Spring)* 2008;16(2):275-84. doi: 10.1038/oby.2007.35 [published Online First: 2008/02/02]
13. Koivusilta LK, West P, Saaristo VM, et al. From childhood socio-economic position to adult educational level - do health behaviours in adolescence matter? A longitudinal study. *BMC public health* 2013;13:711. doi: 10.1186/1471-2458-13-711
14. Kark M, Hjern A, Rasmussen F. Poor school performance is associated with a larger gain in body mass index during puberty. *Acta Paediatr* 2014;103(2):207-13. doi: 10.1111/apa.12471
15. DeGarmo DS, Forgatch MS, Martinez CR, Jr. Parenting of divorced mothers as a link between social status and boys' academic outcomes: unpacking the effects of socioeconomic status. *Child development* 1999;70(5):1231-45. [published Online First: 1999/11/05]

For peer review only - http://bmjopen.bmj.com/site/about/guidelines.xhtml

16. Freeman A, Tyrovolas S, Koyanagi A, et al. The role of socio-economic status in depression: results from the COURAGE (aging survey in Europe). *BMC public health* 2016;16(1):1098. doi: 10.1186/s12889-016-3638-0 [published Online First: 2016/10/21]

17. Scholtens S, Rydell AM, Yang-Wallentin F. ADHD symptoms, academic achievement, self-perception of academic competence and future orientation: a longitudinal study. *Scandinavian journal of psychology* 2013;54(3):205-12. doi: 10.1111/sjop.12042 [published Online First: 2013/03/21]

18. Quek YH, Tam WWS, Zhang MWB, et al. Exploring the association between childhood and adolescent obesity and depression: a meta-analysis. *Obesity reviews : an official journal of the International Association for the Study of Obesity* 2017;18(7):742-54. doi: 10.1111/obr.12535 [published Online First: 2017/04/13]

19. Cole TJ, Lobstein T. Extended international (IOTF) body mass index cut-offs for thinness, overweight and obesity. *Pediatr Obes* 2012;7(4):284-94. doi: 10.1111/j.2047-6310.2012.00064.x

20. Hagman E, Danielsson P, Lindberg L, et al. Paediatric obesity treatment during 14 years in Sweden: Lessons from the Swedish Childhood Obesity Treatment Register-BORIS. *Pediatr Obes* 2020:e12626. doi: 10.1111/ijpo.12626 [published Online First: 2020/02/20]

21. Ludvigsson JF, Otterblad-Olausson P, Pettersson BU, et al. The Swedish personal identity number: possibilities and pitfalls in healthcare and medical research. *European journal of epidemiology* 2009;24(11):659-67. doi: 10.1007/s10654-009-9350-y

22. Ludvigsson JF, Almqvist C, Bonamy AK, et al. Registers of the Swedish total population and their use in medical research. *European journal of epidemiology* 2016;31(2):125-36. doi: 10.1007/s10654-016-0117-y [published Online First: 2016/01/16]

23. Ludvigsson JF, Andersson E, Ekbom A, et al. External review and validation of the Swedish national inpatient register. *BMC public health* 2011;11:450. doi: 10.1186/1471-2458-11-450 [published Online First: 2011/06/11]

24. Wallerstedt SM, Wettermark B, Hoffmann M. The First Decade with the Swedish Prescribed Drug Register - A Systematic Review of the Output in the Scientific Literature. *Basic & clinical pharmacology & toxicology* 2016;119(5):464-69. doi: 10.1111/bcpt.12613 [published Online First: 2016/04/27]

25. Brooke HL, Talback M, Hornblad J, et al. The Swedish cause of death register. *European journal of epidemiology* 2017;32(9):765-73. doi: 10.1007/s10654-017-0316-1 [published Online First: 2017/10/07]

26. UNESCO. International standard classification of education: Isced 1997. Re-edition. Paris: UNESCO Institute for Statistics. 2011

27. Ford AL, Hunt LP, Cooper A, et al. What reduction in BMI SDS is required in obese adolescents to improve body composition and cardiometabolic health? *Archives of disease in childhood* 2010;95(4):256-61. doi: 10.1136/adc.2009.165340 [published Online First: 2009/12/08]

28. Hagman E, Danielsson P, Elimam A, et al. The effect of weight loss and weight gain on blood pressure in children and adolescents with obesity. *Int J Obes (Lond)* 2019 doi: 10.1038/s41366-019-0384-2

29. Nilsson I, Wadeskog A. Utanförskapets Ekonomiska Sociotoper: Socioekonomisk analys på stadsdelsnivå inom ramen för Healthy Cities 2012

30. Finn KE, Seymour CM, Phillips AE. Weight bias and grading among middle and high school teachers. *Br J Educ Psychol* 2019 doi: 10.1111/bjep.12322 [published Online First: 2019/10/28]

31. Nguyen JC, Killcross AS, Jenkins TA. Obesity and cognitive decline: role of inflammation and vascular changes. *Frontiers in neuroscience* 2014;8:375. doi: 10.3389/fnins.2014.00375 [published Online First: 2014/12/06]

32. Tsai C-L, Chen F-C, Pan C-Y, et al. The neurocognitive performance of visuospatial attention in children with obesity. *Frontiers in psychology* 2016;7

For peer review only

For peer review only - http://bmjopen.bmj.com/site/about/guidelines.xhtml

33. Laurent JS, Watts R, Adise S, et al. Associations Among Body Mass Index, Cortical Thickness, and Executive Function in Children. *JAMA Pediatr* 2019 doi: 10.1001/jamapediatrics.2019.4708 [published Online First: 2019/12/10]
34. Sweat V, Yates KF, Migliaccio R, et al. Obese adolescents show reduced cognitive processing speed compared with healthy weight peers. *Childhood Obesity* 2017
35. Cheke LG, Bonnici HM, Clayton NS, et al. Obesity and insulin resistance are associated with reduced activity in core memory regions of the brain. *Neuropsychologia* 2017;96:137-49.
36. Bruehl H, Sweat V, Tirsi A, et al. Obese Adolescents with Type 2 Diabetes Mellitus Have Hippocampal and Frontal Lobe Volume Reductions. *Neuroscience and medicine* 2011;2(1):34-42. doi: 10.4236/nm.2011.21005
37. Spyridaki EC, Simos P, Avgoustinaki PD, et al. The association between obesity and fluid intelligence impairment is mediated by chronic low-grade inflammation. *British Journal of Nutrition* 2014;112(10):1724-34.
38. Mangone A, Yates KF, Sweat V, et al. Cognitive functions among predominantly minority urban adolescents with metabolic syndrome. *Applied Neuropsychology: Child* 2017:1-7.
39. Alosco ML, Spitznagel MB, Strain G, et al. Improved memory function two years after bariatric surgery. *Obesity* 2014;22(1):32-38.
40. Olbers T, Beamish AJ, Gronowitz E, et al. Laparoscopic Roux-en-Y gastric bypass in adolescents with severe obesity (AMOS): a prospective, 5-year, Swedish nationwide study. *Lancet Diabetes Endocrinol* 2017;5(3):174-83. doi: 10.1016/S2213-8587(16)30424-7
41. Biessels GJ, Reagan LP. Hippocampal insulin resistance and cognitive dysfunction. *Nature Reviews Neuroscience* 2015;16(11):660.
42. Latner JD, Stunkard AJ. Getting worse: the stigmatization of obese children. *Obesity research* 2003;11(3):452-6. doi: 10.1038/oby.2003.61
43. Pierce JW, Wardle J. Cause and effect beliefs and self-esteem of overweight children. *Journal of child psychology and psychiatry, and allied disciplines* 1997;38(6):645-50. [published Online First: 1997/10/08]
44. Lindberg L, Ek A, Nyman J, et al. Low grandparental social support combined with low parental socioeconomic status is closely associated with obesity in preschool-aged children: a pilot study. *Pediatr Obes* 2015 doi: 10.1111/ijpo.12049

For peer review only

For peer review only - http://bmjopen.bmj.com/site/about/guidelines.xhtml

Supporting information

S1 Table. International Classification of Diseases (ICD 10th revision) codes and Anatomical Therapeutic Chemical (ATC) classification system codes used.

S2 Table. Proportion of children completing ≥12 years of schooling.

S3 Table. Proportion of individuals who have undergone obesity treatment in childhood who completed ≥12 years of schooling by calendar year and by age at start of obesity treatment (n=3,942).

For peer review only

For peer review only - http://bmjopen.bmj.com/site/about/guidelines.xhtml

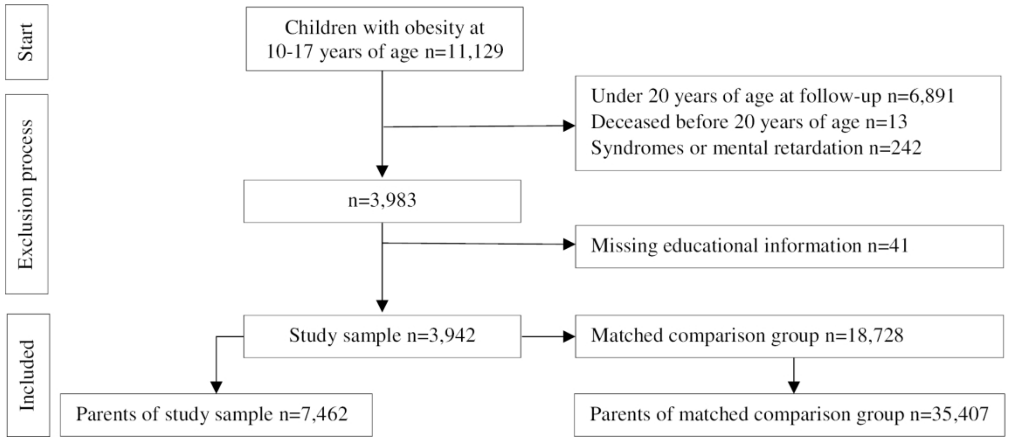

Figure 1

169x73mm (300 x 300 DPI)

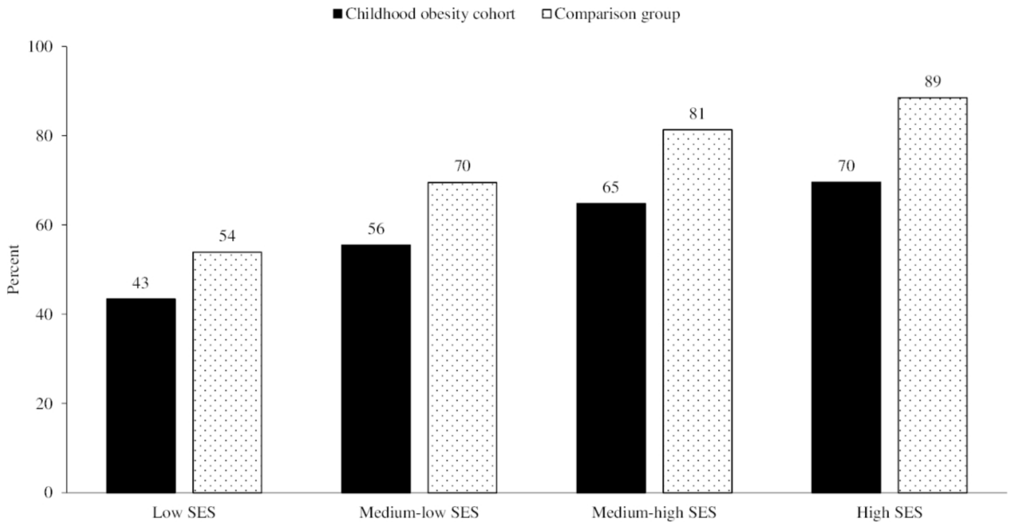

118x60mm (300 x 300 DPI)

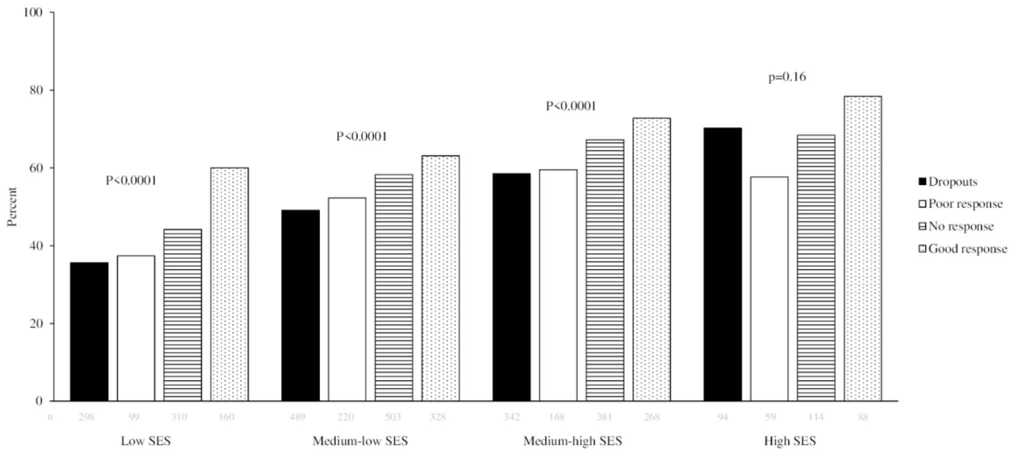

150x66mm (300 x 300 DPI)

S1 Table. International Classification of Diseases (ICD 10th revision) codes and Anatomical Therapeutic Chemical (ATC) classification system codes used.

Condition	ICD-10	ATC Code
Anxiety/sedatives	F40.0-F40.1, F41-F42, F51.4-F51.9, F93.0-F93.2	N05B
Depression/antidepressants	F32-F39, F92	N06A
ADHD/ADD	F90	N06B
Mental retardation	F70-F79	
Genetic syndromes		
Fragile X	Q99.2	
Klinefelter	Q98	
Laurence-Moon-Bardet-Biedl	Q87.8B	
Mb Down	Q90	
Noonan	Q87.1E	
Prader-Willi	Q87.1F	
Silver Russell	Q87.1G	
Turner	Q96	

For peer review only

S2 Table. Proportion of children completing ≥12 years of schooling

	Childhood obesity cohort		Comparison group		
	n	%	n	%	p-value¶
Total	2,236	56.7	13,939	74.4	<0.0001
Sex					
Girl	1,090	59.7	6,843	78.7	<0.0001
Boy	1,146	54.1	7,096	70.8	<0.0001
Migration background					
Nordic	1,695	58.4	10,974	78.1	<0.0001
Non-Nordic	541	52.2	2,965	63.4	<0.0001
ADHD/ADD					
Non-ADHD/ADD	2,032	61.1	13,528	76.5	<0.0001
ADHD/ADD	204	33.1	411	39.4	0.01
Anxiety/depression					
No anxiety/depression	1,902	61.1	12,754	77.0	<0.0001
Anxiety/depression	334	40.2	1,185	54.9	<0.0001
Parental SES					
Low	376	43.4	1,455	53.9	<0.0001
Medium-low	855	55.5	4,187	69.5	<0.0001
Medium-high	751	64.8	5,358	81.3	<0.0001
High	247	69.6	2,848	88.5	<0.0001
Maternal education					
Compulsory school	755	47.7	3,474	61.9	<0.0001
Upper secondary school	945	61.3	5,582	77.9	<0.0001
University degree	506	69.3	4,589	85.3	<0.0001
Paternal education					
Compulsory school	639	50.2	2,775	63.0	<0.0001
Upper secondary school	1,132	59.9	7,085	77.9	<0.0001
University degree	318	66.0	3,333	85.2	<0.0001
Maternal income					
Q1	614	52.3	3,327	68.1	<0.0001
Q2	750	58.6	4,391	75.2	<0.0001
Q3	542	58.2	3,534	77.1	<0.0001
Q4	304	62.6	2,429	81.3	<0.0001
Paternal income					
Q1	556	49.3	2,453	62.6	<0.0001
Q2	410	54.0	2,260	72.3	<0.0001
Q3	549	61.8	3,404	77.6	<0.0001
Q4	572	66.2	5,056	84.5	<0.0001
Maternal occupational status					
No occupation	438	47.8	1,722	58.3	<0.0001
Occupation	1,777	60.0	12,005	77.9	<0.0001
Paternal occupational status					
No occupation	363	45.4	1,543	58.7	<0.0001
Occupation	1,736	60.5	11,703	78.5	<0.0001

Abbreviations: SES, socioeconomic status; Q, quartile. ¶Performed with chi-square.
1 EUR ≈ 9.83 SEK December 31st 2017. Q1: <18,652 EUR; Q2: 18,652 to 25,025 EUR; Q3: 25,026 to 32,309 EUR; Q4: >32,309 EUR.

For peer review only

S3 Table. Proportion of individuals who have undergone obesity treatment in childhood who completed ≥12 years of schooling by calendar year and by age at start of obesity treatment (n=3,942).

	Individuals completing ≥12 years of schooling	
	n	%
Calendar year at start of obesity treatment		
1995-2001	229	62.6
2002-2008	1,262	58.1
2009-2014	745	53.1
Age at start of obesity treatment		
10 - 12.99	960	59.0
13 - 16.99	1,276	55.1

For peer review only

STROBE Statement—Checklist of items that should be included in reports of ***cohort studies***

	Item No	Recommendation	Page No
**Title and abstract**	1	(*a*) Indicate the study's design with a commonly used term in the title or the abstract	1-2
		(*b*) Provide in the abstract an informative and balanced summary of what was done and what was found	2-3
**Introduction**			
Background/rationale	2	Explain the scientific background and rationale for the investigation being reported	4
Objectives	3	State specific objectives, including any prespecified hypotheses	4.5
**Methods**			
Study design	4	Present key elements of study design early in the paper	5
Setting	5	Describe the setting, locations, and relevant dates, including periods of recruitment, exposure, follow-up, and data collection	5-9
Participants	6	(*a*) Give the eligibility criteria, and the sources and methods of selection of participants. Describe methods of follow-up	5
		(*b*) For matched studies, give matching criteria and number of exposed and unexposed	5
Variables	7	Clearly define all outcomes, exposures, predictors, potential confounders, and effect modifiers. Give diagnostic criteria, if applicable	7-9
Data sources/ measurement	8*	For each variable of interest, give sources of data and details of methods of assessment (measurement). Describe comparability of assessment methods if there is more than one group	7-9
Bias	9	Describe any efforts to address potential sources of bias	7-9
Study size	10	Explain how the study size was arrived at	5
Quantitative variables	11	Explain how quantitative variables were handled in the analyses. If applicable, describe which groupings were chosen and why	9-10
Statistical methods	12	(*a*) Describe all statistical methods, including those used to control for confounding	9-10
		(*b*) Describe any methods used to examine subgroups and interactions	
		(*c*) Explain how missing data were addressed	
		(*d*) If applicable, explain how loss to follow-up was addressed	
		(*e*) Describe any sensitivity analyses	
**Results**			
Participants	13*	(a) Report numbers of individuals at each stage of study—eg numbers potentially eligible, examined for eligibility, confirmed eligible, included in the study, completing follow-up, and analysed	12 + fig
		(b) Give reasons for non-participation at each stage	
		(c) Consider use of a flow diagram	
Descriptive data	14*	(a) Give characteristics of study participants (eg demographic, clinical, social) and information on exposures and potential confounders	11
		(b) Indicate number of participants with missing data for each variable of interest	
		(c) Summarise follow-up time (eg, average and total amount)	
Outcome data	15*	Report numbers of outcome events or summary measures over time	12

For peer review only - http://bmjopen.bmj.com/site/about/guidelines.xhtml

Main results	16	(*a*) Give unadjusted estimates and, if applicable, confounder-adjusted estimates and their precision (eg, 95% confidence interval). Make clear which confounders were adjusted for and why they were included<br>(*b*) Report category boundaries when continuous variables were categorized<br>(*c*) If relevant, consider translating estimates of relative risk into absolute risk for a meaningful time period	12-17
Other analyses	17	Report other analyses done—eg analyses of subgroups and interactions, and sensitivity analyses	12-17
**Discussion**			
Key results	18	Summarise key results with reference to study objectives	18
Limitations	19	Discuss limitations of the study, taking into account sources of potential bias or imprecision. Discuss both direction and magnitude of any potential bias	20-21
Interpretation	20	Give a cautious overall interpretation of results considering objectives, limitations, multiplicity of analyses, results from similar studies, and other relevant evidence	18-21
Generalisability	21	Discuss the generalisability (external validity) of the study results	21
**Other information**			
Funding	22	Give the source of funding and the role of the funders for the present study and, if applicable, for the original study on which the present article is based	22-23

*Give information separately for exposed and unexposed groups.

Note: An Explanation and Elaboration article discusses each checklist item and gives methodological background and published examples of transparent reporting. The STROBE checklist is best used in conjunction with this article (freely available on the Web sites of PLoS Medicine at http://www.plosmedicine.org/, Annals of Internal Medicine at http://www.annals.org/, and Epidemiology at http://www.epidem.com/). Information on the STROBE Initiative is available at http://www.strobe-statement.org.

For peer review only

BMJ Open

Obesity in childhood, socioeconomic status, and completion of 12 or more school years: a prospective cohort study

Journal:	*BMJ Open*
Manuscript ID	bmjopen-2020-040432.R1
Article Type:	Original research
Date Submitted by the Author:	03-Nov-2020
Complete List of Authors:	Lindberg, Louise; Karolinska Institutet, Dept. Clinical Science, Intervention and Technology<br>Persson, Martina; Karolinska Institute, Department of Medicine, Clinical Epidemiology, Karolinska Institutet;<br>Danielsson, Pernilla; Karolinska Institutet, Dept. Clinical Science, Intervention and Technology<br>Hagman, Emilia; Karolinska Institutet, Dept. Clinical Science, Intervention and Technology<br>Marcus, Claude; Karolinska Institutet, Dept. Clinical Science, Intervention and Technology
**Primary Subject Heading**:	Paediatrics
Secondary Subject Heading:	Epidemiology, Nutrition and metabolism, Public health
Keywords:	Paediatric endocrinology < DIABETES & ENDOCRINOLOGY, EPIDEMIOLOGY, Paediatric endocrinology < PAEDIATRICS, PUBLIC HEALTH

SCHOLARONE™
Manuscripts

I, the Submitting Author has the right to grant and does grant on behalf of all authors of the Work (as defined in the below author licence), an exclusive licence and/or a non-exclusive licence for contributions from authors who are: i) UK Crown employees; ii) where BMJ has agreed a CC-BY licence shall apply, and/or iii) in accordance with the terms applicable for US Federal Government officers or employees acting as part of their official duties; on a worldwide, perpetual, irrevocable, royalty-free basis to BMJ Publishing Group Ltd ("BMJ") its licensees and where the relevant Journal is co-owned by BMJ to the co-owners of the Journal, to publish the Work in this journal and any other BMJ products and to exploit all rights, as set out in our licence.

The Submitting Author accepts and understands that any supply made under these terms is made by BMJ to the Submitting Author unless you are acting as an employee on behalf of your employer or a postgraduate student of an affiliated institution which is paying any applicable article publishing charge ("APC") for Open Access articles. Where the Submitting Author wishes to make the Work available on an Open Access basis (and intends to pay the relevant APC), the terms of reuse of such Open Access shall be governed by a Creative Commons licence – details of these licences and which Creative Commons licence will apply to this Work are set out in our licence referred to above.

Other than as permitted in any relevant BMJ Author's Self Archiving Policies, I confirm this Work has not been accepted for publication elsewhere, is not being considered for publication elsewhere and does not duplicate material already published. I confirm all authors consent to publication of this Work and authorise the granting of this licence.

For peer review only

Obesity in childhood, socioeconomic status, and completion of 12 or more school years: a prospective cohort study

Louise Lindberg[1], Martina Persson[2,3,4], Pernilla Danielsson[1], Emilia Hagman[1*], Claude Marcus[1]

[1]Department of Clinical Science, Intervention and Technology, Division of Pediatrics, Karolinska Institutet, Stockholm, Sweden

[2]Department of Medicine Solna, Clinical Epidemiology Unit, Karolinska Institutet, Stockholm, Sweden

[3]Department of Diabetes and Endocrinology, Sachsska Children's Hospital Södersjukhuset, Stockholm, Sweden

[4]Department of Clinical Science and Education, Södersjukhuset, Karolinska Institutet, Stockholm, Sweden

*Corresponding author e-mail: emilia.hagman @ki.se (EH)

Other contributing authors e-mail: louise.lindberg@ki.se; martina.persson@ki.se; pernilla.danielsson.liljeqvist@ki.se; claude.marcus@ki.se

Keywords: Pediatric obesity, schools, epidemiology, social class, cohort study

Word count: 3722

For peer review only

For peer review only - http://bmjopen.bmj.com/site/about/guidelines.xhtml

Abstract

Objectives: Children with obesity achieve lower educational level compared with normal-weight peers. Parental socioeconomic status (SES) impacts both a child's academic achievement and risk of obesity. The degree to which the association between obesity and education depend on parental SES is unclear. Therefore, the primary aim is to investigate if individuals with obesity in childhood are less likely to complete ≥12 years of schooling, independently of parental SES. The secondary aim is to study how weight loss, level of education, and parental SES are associated.

Design: Nationwide prospective cohort study.

Setting: Swedish national register data.

Participants: Children aged 10-17 years, recorded in the Swedish Childhood Obesity Treatment Register (BORIS), and aged 20 years or older at follow-up were included (n=3,942). A comparison group was matched by sex, year of birth, and living area (n=18,728). Parental SES was based on maternal and paternal level of education, income, and occupational status.

Primary outcome measure: Completion of ≥12 years of schooling was analysed with conditional logistic regression, and adjusted for group, migration background, ADHD/ADD, anxiety/depression, and parental SES.

Results: Among those with obesity in childhood, 56.7% completed ≥12 school years compared with 74.4% in the comparison group (p<0.0001). High parental SES compared with low SES was strongly associated with attained level of education in both children with and without obesity, adjusted odds ratio (^{a}OR) [99% confidence interval (CI)]=5.40 [4.45 to 6.55]. However, obesity in childhood remains a strong risk factor of not completing ≥12

For peer review only - http://bmjopen.bmj.com/site/about/guidelines.xhtml

school years, independently of parental SES, [a]OR=0.57 [0.51 to 0.63]. Successful obesity treatment increased the odds of completing ≥12 years in school even when taking parental SES into account, [a]OR=1.34 [1.04 to 1.72].

Conclusions: Individuals with obesity in childhood have lower odds of completing ≥12 school years, independently of parental SES. Optimized obesity treatment may improve school results in this group.

Strengths and limitation of this study

- In this prospective cohort study, we have been able to investigate the level of education among a large number of individuals who have obesity in childhood (n= 3,942) in comparison with a matched group (n=18,728).
- The study design of using longitudinal data from several national registers provided the opportunity to control for important confounding factors, such as neuropsychiatric disorders, anxiety, depression, and family socioeconomic status.
- Factors such as free education, school lunches, and students' health care may have an impact on the generalizability of our data to other populations.

For peer review only - http://bmjopen.bmj.com/site/about/guidelines.xhtml

Introduction

During the last 40 years, the prevalence of childhood obesity has increased exponentially in many parts of the world.[1] Childhood obesity is associated with increased risks of somatic morbidity and risk of premature death in adulthood.[2-5] Psychosocial maladjustment, anxiety and depression are also prevalent[6 7] and may contribute to the obesity-related long-term morbidity and mortality.[5] Most studies report that children with obesity more often have lower school grades and reach a lower level of education compared with normal-weight peers,[8-10] but in a recent study, only girls with obesity were affected.[11] We have previously confirmed the association between obesity and lower attained level of education among both girls and boys.[9] We also found that successful obesity treatment was positively correlated with completing ≥12 school years, although without taking socioeconomic status (SES) into account.[9]

Low parental SES is a well-established risk factor for both childhood obesity and poorer academic achievement.[12] In particular, parental education has been demonstrated to influence the child's performance at school.[13-15] In addition, depression[16] and neuropsychiatric disorders, including Attention Deficit Disorder with or without Hyperactivity (ADHD/ADD),[17] may adversely affect school results. Depression and neuropsychiatric disorders are more common in children with obesity compared to the general population.[9 18] Investigating the impact of obesity alone on attained level of education requires that these and other confounders are considered. The primary aim of this study was to disentangle the association of childhood obesity and parental SES on completed level of education. The

For peer review only - http://bmjopen.bmj.com/site/about/guidelines.xhtml

secondary aim was to study if positive effects of weight loss on attained level of education is affected by parental SES.

Methods

Study population

This prospective cohort study included children with obesity,[19] aged 10-17 years at the start of obesity treatment (December 1994 to December 2015), and aged 20 years or older and living in Sweden at follow-up (December 31st 2017; n=3,942). Data on subjects receiving treatment for childhood obesity was collected from the Swedish Childhood Obesity Treatment Register (BORIS). BORIS has been thoroughly described elsewhere,[20] but in short: The main purpose of the register is quality assessment and long-term evaluation of childhood obesity treatment. Treatment is individualized and consists primarily of lifestyle modification (i.e. diet and physical activity).

A comparison group from the general population was randomly identified using the Swedish Total Population Register and matched by sex, year of birth, and living area at the year obesity treatment was initiated (n=18,728). Using density matching without replacement, five individuals were matched to each individual from the childhood obesity cohort. Siblings of children registered in the childhood obesity cohort were excluded from the comparison group. Children with a diagnosis of mental retardation or genetic syndromes were excluded from both the childhood obesity cohort and the comparison group (Fig 1 and S1 Table).

For peer review only - http://bmjopen.bmj.com/site/about/guidelines.xhtml

Families were informed in written or verbal about data collection in the Swedish Childhood Obesity Treatment Register. Post an opt-out approval by parents/guardians, data of the children's weight and height were recorded by the local health care provider during treatment visits. There was no data of weight and height of individuals in the comparison group. However, less than 1% of the individuals in the comparison group were found in the National Patient Register with a diagnosis of obesity in childhood. In Sweden, health care is free of charge for children and adolescents until 18 years of age. The study was approved by the regional Ethics Committee in Stockholm, Sweden (No. 2016/922-31/1).

Data sources

Using the Swedish identity number, which is unique to each resident in Sweden, data from several national registers were linked.[21] Data on education, income, and occupational status were obtained from the Longitudinal Integration Database for Health Insurance and Labour Market Studies. Information on migration background for children and their parents was obtained from the Swedish Total Population Register.[22] Both these registers are held by Statistics Sweden, a governmental agency that collects and provide official statistics (www.scb.se/en).

Data on diagnoses of mental retardation, genetic syndromes, ADHD/ADD, anxiety, and depression were identified based on codes according the International Classification of Diseases (10th revision; S1 Table) and retrieved from the National Patient Register.[23] Information on prescriptions of psychostimulant drugs for ADHD/ADD as well as antidepressants and tranquilizers for anxiety and depression were identified using the Anatomical Therapeutic Chemical classification system (S1 Table) retrieved from the

For peer review only - http://bmjopen.bmj.com/site/about/guidelines.xhtml

Swedish Prescribed Drug Register.[24] Data on deaths were retrieved from the Cause of Death Register.[25] These registers are held and were linked by the governmental agency the National Board of Health and Welfare (www.socialstyrelsen.se/english).

Definition of outcome

The main outcome was defined as completion of ≥12 years of schooling, and based on the International Standard Classification of Education.[26] In Sweden, children start school at the age of 6 or 7 years and attend compulsory school for 9 years. Upper secondary school includes three additional years of schooling and provides the requirements to attend higher education. Usually students graduate from upper secondary school at 18 or 19 years of age. All education in Sweden is free of charge. Students in compulsory and upper secondary school are provided school lunches and health care at schools, also free of charge.

Definition of exposure variables

Degree of obesity in children from BORIS was assessed with Body Mass Index Standard Deviation Score (BMI SDS), which is standardized by sex and age and used to measure degree of obesity in growing children.[19] Baseline measures (continuous) were used for the variable BMI SDS at start of treatment. Response to obesity treatment was based on the change of BMI SDS from the first to the last clinical visit and categorized into four groups:[3,27] good response, a reduction of BMI SDS by 0.25 units or more; no response, a change of BMI SDS by +/- 0.24 units; poor response, an increase of BMI SDS by 0.25 units or more; and dropouts, children with less than one year between their first and last measure or without clinical follow-up after their first registered visit.

For peer review only - http://bmjopen.bmj.com/site/about/guidelines.xhtml

Parental SES was based on maternal and paternal level of education, income, and occupational status at the year the child turned 15 years, which is about the same time as the child starts upper secondary school. In case of the child was adopted, the SES of adoptive parents was used (childhood obesity cohort n=24 and comparison group n=164). The rationale of treating SES as a composite variable was to capture more of the social context and a potential inequality embedded there. Thus, by taking three variables into account instead of one, we get a more wide and robust measure of SES. Maternal and paternal educational level was categorized into compulsory school, upper secondary school, or university degree. Annual disposable income was used to reflect maternal and paternal economic capacity. The annual disposable income includes all taxable (direct labour income, capital gains from shares etc.) and tax-free income (housing and child benefits, student aid etc.), minus final tax and other negative transfers such as capital loss from shares and properties. Disposable income from different years was converted to 2017 prices using the Consumer Price Index for Sweden provided by Statistics Sweden (www.scb.se/en). Income was categorized into quartiles based on data from the parents in the comparison group**.** No occupation was defined as either unemployment 6 months or more, or income from long-term sick leave exceeding any income from the individual's gross salary. Individuals considered to have an occupation included those registered as employed or having an income from student grants/loan equivalent to full-time studies for at least one semester. Parental SES was weighted according to maternal and paternal education (0, 1, 2), income (0, 1, 2, 3), and occupational status (0, 1). The mean parental SES score (i.e. the sum of the mother's and father's all SES indicators divided by two) was applied to their child. The SES-variable was

For peer review only - http://bmjopen.bmj.com/site/about/guidelines.xhtml

categorized into four levels; low SES (0 to 1.5 points), medium-low SES (2 to 3 points), medium-high SES (3.5 to 4.5 points), and high SES (5 to 6 points).

The prevalence of both ADHD/ADD[9] and depression[18] is higher in children with obesity and may negatively influence attained level of education.[9 16 17] ADHD/ADD and anxiety/depression in children were identified based on diagnosis or dispensed prescribed medication (S1 Table).

Definition of covariates

The migration background of children in Sweden may impact school achievements and is therefore an important factor to control for in the analyses.[9 14] Migration background was categorized as Nordic or non-Nordic. Nordic was defined as born in a Nordic country (Sweden, Norway, Denmark, Finland, or Iceland) with one or two parents born in the Nordic. Children were classified as non-Nordic if born outside the Nordic or born in the Nordic with two parents born outside the Nordic. Other covariates included sex and age (continuous) at start of obesity treatment.

Patient and public involvement

No patient involved.

Statistical analysis

Descriptive statistics are presented as means and confidence intervals (CI), medians and interquartile ranges (IQR), or frequencies and percentages. Conditional logistic regression

For peer review only - http://bmjopen.bmj.com/site/about/guidelines.xhtml

was used to calculate odds ratios (OR's) with 99% CI for the main outcome, i.e. completing ≥12 school years. Conditional logistic regression was used above ordinary logistic regression as the childhood obesity cohort and the comparison group were matched by several variables. Independent variables included in the adjusted analyses were migration background, ADHD/ADD, anxiety/depression, parental SES, and group (childhood obesity cohort or comparison group) in analyses including both groups, otherwise stratified by group. Interaction between childhood obesity and parental SES was tested. The association of each SES indicator on the odds of completing ≥12 school years was also analysed separately for mothers and fathers. Sensitivity analyses excluding individuals with a non-Swedish background (defined as child born in Sweden with at least one parent also born in Sweden), or ADHD/ADD, or anxiety/depression were performed.

Secondary analyses were performed within the childhood obesity cohort to examine associations between patient characteristics and completed educational level. In ordinary logistic regressions, odds were adjusted for sex, migration background, parental SES, age and BMI SDS at start of treatment, treatment response, ADHD/ADD, and anxiety/depression. We tested for possible interaction between parental SES and treatment response for the odds of completing ≥12 years of schooling.

As missing data on parental SES was rare in both groups (Table 1), records with missing data were excluded from the analyses, i.e. data were not imputed. P-values <0.01 were considered statistically significant. All analyses were performed using SAS statistical software (version 9.4, Cary, NC, USA).

For peer review only - http://bmjopen.bmj.com/site/about/guidelines.xhtml

Table 1. Characteristics of the participants (n=22,670)

	Childhood obesity cohort (n=3,942)		Comparison group (n=18,728)	
	n	%	n	%
Female sex	1,825	46.3	8,701	46.5
Nordic	2,905	73.7	14,048	75.0
Age at end of follow-up (years)	23.4*	21.4-26.3*	23.4*	21.5-26.3*
ADHD/ADD	617	15.7	1,044	5.6
Anxiety/Depression	831	21.1	2,158	11.5
Parental SES				
Low	867	22.0	2,700	14.4
Medium-low	1,540	39.1	6,023	32.1
Medium-high	1,159	29.4	6,587	35.2
High	355	9.0	3,218	17.2
Missing	21	0.5	200	1.1
Maternal education				
Compulsory school	1,582	40.1	5,615	30.0
Upper secondary school	1,541	39.1	7,167	38.3
University degree	730	18.5	5,383	28.7
Missing	89	2.3	563	3.0
Paternal education				
Compulsory school	1,273	32.3	4,402	23.5
Upper secondary school	1,890	48.0	9,095	48.6
University degree	482	12.2	3,912	20.9
Missing	297	7.5	1,319	7.0
Maternal income				
Q1	1,173	29.8	4,887	26.1
Q2	1,281	32.5	5,843	31.2
Q3	931	23.6	4,585	24.5
Q4	486	12.3	2,988	15.9
Missing	71	1.8	425	2.3
Paternal income				
Q1	1,127	28.6	3,917	20.9
Q2	760	19.3	3,128	16.7
Q3	889	22.5	4,389	23.4
Q4	864	21.9	5,986	32.0
Missing	302	7.7	1,308	7.0
Maternal occupational status				
Occupation	2,963	75.2	15,405	82.2
No occupation	916	23.2	2,956	15.8
Missing	63	1.6	367	2.0
Paternal occupational status				
Occupation	2,868	72.8	14,906	79.6
No occupation	800	20.3	2,627	14.0
Missing	274	6.9	1,195	6.4

Data are n % if not else stated. Abbreviation: SES, socioeconomic status; Q, quartile.
*Median with interquartile ranges

For peer review only

For peer review only - http://bmjopen.bmj.com/site/about/guidelines.xhtml

Results

In total, 3,942 individuals in the childhood obesity cohort and 18,728 individuals in the comparison group were included in the study (Table 1). In both groups, 46% of the participants were girls and the median age at follow-up was 23.4 years. The proportions of individuals of Nordic origin were similar in both groups (73.7% vs. 75.0%; p=0.02). Despite the groups being matched for living area, a greater proportion of children with obesity grew up in households with low SES compared with the comparison group (22.0% vs. 14.4%; p<0.0001).

Parental SES, childhood obesity and the child's attained level of education

In the childhood obesity cohort, 56.7% completed ≥12 years in school, compared with 74.4% in the comparison group. Girls more frequently completed ≥12 years of schooling than boys in both groups (S2 Table).

Higher parental SES was positively associated with completion of ≥12 years of schooling in both the childhood obesity cohort and in the comparison group (Fig 2). The odds were more than five times higher among children growing up in high SES households compared with those growing up in low SES households (Table 2). However, even when taking parental SES and other risk factors into account, individuals in the childhood obesity cohort were almost half as likely to complete ≥12 school years compared with those in the comparison group (Table 2). The adjusted odds ratio ([a]OR) [99% CI] to complete ≥12 years of schooling stratified by parental SES, when comparing the childhood obesity cohort with the comparison

For peer review only - http://bmjopen.bmj.com/site/about/guidelines.xhtml

group, showed a trend towards lower OR in the higher level of SES: low parental SES = 0.69 [0.50 to 0.95], p=0.0026; medium-low parental SES = 0.59 [0.48 to 0.72], p<0.0001; medium-high parental SES = 0.46 [0.35 to 0.60], p<0.0001; high parental SES = 0.27 [0.14 to 0.54], p<0.0001. P-value for interaction test for childhood obesity and parental SES reaches a p of 0.0015.

The association of parental SES on school performance was more pronounced in the comparison group than in the childhood obesity cohort. For example, stratified analyses show that adjusted OR of completing ≥12 years in school in low SES compared with high SES in the comparison group was 0.17 (99% CI 0.14 to 0.21, p<0.0001) and in the childhood obesity cohort 0.31 (99% CI 0.22 to 0.45, p<0.0001). Regardless of how we divide the childhood obesity population (into age- or calendar year at start of obesity treatment, SES sub-scores), large differences between the two groups remain (Table 2 and S3 Table).

For peer review only

For peer review only - http://bmjopen.bmj.com/site/about/guidelines.xhtml

Table 2. Crude and adjusted odds ratio (99% CI); p-value of subjects completing ≥12 years of schooling

	Crude estimates	Model 1, n=22 449	Model 2, n=20 610	Model 3, n=20 691	Model 4, n=20 885	Model 5, n=20 420
Childhood obesity cohort vs. comparison group	0.44 (0.40 to 0.48); <0.0001	0.57 (0.51 to 0.63); <0.0001	0.55 (0.50 to 0.62); <0.0001	0.54 (0.48 to 0.61); <0.0001	0.53 (0.48 to 0.59); <0.0001	0.59 (0.52 to 0.66); <0.0001
Non-Nordic vs. Nordic	0.70 (0.63 to 0.78); <0.0001	0.85 (0.75 to 0.97); 0.001	0.77 (0.67 to 0.88); <0.0001	0.87 (0.76 to 0.99); 0.0068	0.81 (0.71 to 0.92); <0.0001	0.97 (0.84 to 1.12); 0.5687
ADHD/ADD vs. non-ADHD/ADD	0.19 (0.17 to 0.23); <0.0001	0.28 (0.24 to 0.33); <0.0001	0.27 (0.22 to 0.33); <0.0001	0.27 (0.22 to 0.32); <0.0001	0.26 (0.22 to 0.31); <0.0001	0.28 (0.23 to 0.34); <0.0001
Anxiety/depression vs. no anxiety/depression	0.31 (0.27 to 0.35); <0.0001	0.39 (0.34 to 0.45); <0.0001	0.36 (0.31 to 0.41); <0.0001	0.38 (0.33 to 0.44); <0.0001	0.39 (0.33 to 0.44); <0.0001	0.36 (0.31 to 0.42); <0.0001
Parental SES						
Medium-low vs. low parental SES	1.74 (1.54 to 1.97); <0.0001	1.69 (1.48 to 1.93); <0.0001				
Medium-high vs. low parental SES	3.33 (2.92 to 3.81); <0.0001	2.99 (2.59 to 3.45); <0.0001				
High vs. low parental SES	6.21 (5.19 to 7.44); <0.0001	5.40 (4.45 to 6.55); <0.0001				
Maternal education						
Upper secondary school vs. compulsory school	1.88 (1.69 to 2.08); <0.0001		1.63 (1.45 to 1.83); <0.0001			1.51 (1.34 to 1.71); <0.0001
University degree vs. compulsory school	3.23 (2.86 to 3.66); <0.0001		2.45 (2.12 to 2.83); <0.0001			2.17 (1.87 to 2.53); <0.0001
Paternal education						
Upper secondary school vs. compulsory school	1.88 (1.69 to 2.09); <0.0001		1.58 (1.40 to 1.77); <0.0001			1.42 (1.26 to 1.60); <0.0001
University degree vs. compulsory school	3.07 (2.66 to 3.55); <0.0001		2.09 (1.77 to 2.45); <0.0001			1.81 (1.53 to 2.15); <0.0001
Maternal income						
Q2 vs. Q1	1.28 (1.15 to 1.43); <0.0001			1.25 (1.10 to 1.41); <0.0001		1.07 (0.93 to 1.23); 0.2065
Q3 vs. Q1	1.45 (1.28 to 1.63); <0.0001			1.35 (1.17 to 1.55); <0.0001		1.05 (0.90 to 1.23); 0.4224
Q4 vs. Q1	1.86 (1.60 to 2.15); <0.0001			1.52 (1.28 to 1.81); <0.0001		1.07 (0.88 to 1.28); 0.3831
Paternal income						
Q2 vs. Q1	1.36 (1.19 to 1.55); <0.0001			1.31 (1.13 to 1.51); <0.0001		1.07 (0.91 to 1.26); 0.2740
Q3 vs. Q1	1.90 (1.67 to 2.17); <0.0001			1.70 (1.48 to 1.96); <0.0001		1.24 (1.05 to 1.46); 0.0009
Q4 vs. Q1	2.95 (2.58 to 3.36); <0.0001			2.47 (2.13 to 2.86); <0.0001		1.58 (1.33 to 1.88); <0.0001
Maternal occupational status						
Occupation vs. no occupation	2.15 (1.92 to 2.40); <0.0001				1.72 (1.51 to 1.96); <0.0001	1.38 (1.19 to 1.61); <0.0001
Paternal occupational status						
Occupation vs. no occupation	2.24 (1.99 to 2.52); <0.0001				1.79 (1.57 to 2.04); <0.0001	1.41 (1.20 to 1.65); <0.0001

Abbreviations: CI, confidence interval; SES, socioeconomic status.
Model 1: Variables included were group (childhood obesity cohort vs. comparison group), migration background, ADHD/ADD, anxiety/depression, and parental SES
Model 2: Variables included were group, migration background, ADHD/ADD, anxiety/depression, maternal-, and paternal education
Model 3: Variables included were group, migration background, ADHD/ADD, anxiety/depression, maternal-, and paternal income
Model 4: Variables included were group, migration background, ADHD/ADD, anxiety/depression, maternal-, and paternal occupational status.
Model 5: Variables included were group, migration background, ADHD/ADD, anxiety/depression, maternal-, and paternal education, income, and occupational status.

For peer review only

For peer review only - http://bmjopen.bmj.com/site/about/guidelines.xhtml

Of note, having a non-Nordic-, compared to a Nordic background, was associated with reduced odds to complete 12 or more years of schooling (Table 2). This was however not observed in boys in the childhood obesity cohort (p=0.68). Further, excluding individuals with a non-Swedish background, or ADHD/ADD, or anxiety/depression in sensitivity analyses, did not alter the association between childhood obesity and attained level of education. For example, when excluding individuals with a non-Swedish background, the [a]OR to complete ≥12 years in school for the obesity cohort vs. the comparison group was 0.55 [0.48 to 0.63], p <0.0001.

Degree of obesity and treatment response on completed educational level in children with obesity

In the childhood obesity cohort, the mean age at start of treatment was 13.5 years (99% CI: 13.45 to 13.62) and the mean BMI SDS was +2.91 (99% CI: 2.89 to 2.92). The median treatment duration was 2.83 years (IQR: 1.86 to 4.42) and the mean treatment response, calculated using BMI SDS from the first to the last clinical visit, was -0.13 (99% CI: -0.15 to -0.10, n=2,709, 68.7%, dropouts excluded). At baseline, 50.7% had obesity and 49.3% had morbid obesity. At last measured weight and height, 33.4% had obesity and 47.2% had morbid obesity, while 19.4% of the children no longer had obesity (dropouts excluded).

A greater degree of obesity at start of treatment lowered the odds of completing ≥12 years of schooling, [a]OR [99% CI] = 0.51 [0.40 to 0.64], p<0.0001, per one unit increase in BMI SDS, while age at start of obesity treatment did not influence the outcome (Table 3). Treatment response was categorized as good response (n=847), no response (n=1 315), poor response (n=547), and dropouts (n=1,233). Of those with good treatment response, 67% completed

For peer review only - http://bmjopen.bmj.com/site/about/guidelines.xhtml

268 ≥12 years in school compared with 58%, 52%, and 50% in the groups with no or poor response, and dropouts, respectively ($p<0.0001$). Within all SES groups, except for high SES, greater treatment response resulted in higher odds of completing ≥12 years in school (Fig 3). Dropouts were less likely to complete ≥12 years in school compared with non-responders (Table 3) but this was not observed in the high SES group (Fig 3). Duration of treatment differed between children with good and poor treatment response (3.5 vs. 4.0 years; $p<0.0001$). In the childhood obesity cohort, the association between parental SES and odds of completing ≥12 years of schooling was not modified by treatment response ($p=0.603$).

For peer review only

Table 3. Crude and adjusted odds ratios of completing ≥12 years of schooling in the childhood obesity cohort

	**Odds ratio (99% CI) performed with ordinary logistic regression; p value**		
	Crude estimates	Model 1, n=3 921	Model 2, n=3 575
Sex (girls vs. boys)	1.26 (1.06 to 1.48); 0.0004	1.30 (1.08 to 1.56); 0.0002	1.29 (1.06 to 1.56); 0.0008
Migration background (non-Nordic vs. Nordic)	0.78 (0.65 to 0.94); 0.0006	0.74 (0.60 to 0.91); 0.0002	0.69 (0.55 to 0.87); <0.0001
ADHD/ADD (yes vs. no)	0.31 (0.25 to 0.40); <0.0001	0.35 (0.27 to 0.45); <0.0001	0.33 (0.26 to 0.44); <0.0001
Anxiety/depression (yes vs. no)	0.43 (0.35 to 0.53); <0.0001	0.45 (0.36 to 0.57); <0.0001	0.43 (0.34 to 0.55); <0.0001
BMI SDS at start of treatment	0.47 (0.38 to 0.58); <0.0001	0.51 (0.40 to 0.64); <0.0001	0.48 (0.38 to 0.62); <0.0001
Age at start of treatment	0.96 (0.93 to 1.01); 0.0234	1.01 (0.96 to 1.06); 0.60	1.01 (0.96 to 1.06); 0.805
Treatment response*			
Good response vs. no response	1.46 (1.15 to 1.85); <0.0001	1.34 (1.04 to 1.72); 0.0031	1.28 (0.98 to 1.67); 0.0182
Poor response vs. no response	0.78 (0.60 to 1.02); 0.0167	0.72 (0.54 to 0.96); 0.0028	0.71 (0.53 to 0.96); 0.003
Dropouts vs. no response	0.71 (0.58 to 0.87); <0.0001	0.69 (0.55 to 0.86); <0.0001	0.68 (0.54 to 0.87); <0.0001
Parental SES			
Medium-low vs. low parental SES	1.63 (1.31 to 2.03); <0.0001	1.56 (1.24 to 1.98); <0.0001	
Medium-high vs. low parental SES	2.40 (1.90 to 3.05); <0.0001	2.19 (1.69 to 2.83); <0.0001	
High vs. low parental SES	2.99 (2.11 to 4.22); <0.0001	2.83 (1.95 to 4.12); <0.0001	
Maternal education			
Upper secondary school vs. compulsory school	1.74 (1.44 to 2.09); <0.0001		1.57 (1.27 to 1.94); <0.0001
University degree vs. compulsory school	2.47 (1.94 to 3.16); <0.0001		2.23 (1.67 to 2.98); <0.0001
Paternal education			
Upper secondary school vs. compulsory school	1.48 (1.23 to 1.79); <0.0001		1.26 (1.03 to 1.55); 0.004
University degree vs. compulsory school	1.92 (1.44 to 2.56); <0.0001		1.41 (1.01 to 1.95); 0.0076

Abbreviations: CI, confidence interval; BMI SDS, BMI standard deviation score; SES, socioeconomic status.
* Good response=decrease of BMI SDS ≥ 0.25 units; No response=BMI SDS +/- 0.24 units; Poor response=increase of BMI SDS ≥ 0.25 units; Dropouts=no follow-up measure or less than one year between their first and last measure.
Model 1: Variables included were sex, migration background, ADHD/ADD, anxiety/depression, BMI SDS- and age at start of treatment, treatment response, and parental SES.
Model 2: Variables included were sex, migration background, ADHD/ADD, anxiety/depression, BMI SDS- and age at start of treatment, treatment response, maternal-, and paternal education.

For peer review only

For peer review only - http://bmjopen.bmj.com/site/about/guidelines.xhtml

Discussion

In this prospective cohort study, we have compared level of education among individuals recorded in the Swedish Childhood Obesity Treatment Register, and a comparison group matched by sex, year of birth, and living area. Individuals in the childhood obesity cohort were half as likely to complete 12 or more years of schooling, independently of parental socioeconomic status (SES).

Among individuals from high SES families, those in the childhood obesity cohort were approximately one third as likely to complete ≥12 years of schooling as individuals from high SES families in the comparison group. Furthermore, our results indicate that parental SES was more important to complete ≥12 school years in the comparison group than in the childhood obesity cohort. An association between obesity and impaired academic achievements has been demonstrated before.[8 9] To which extent other psychosocial factors contribute to this finding has not been evaluated. It is well established that low parental SES,[13-15] immigrant background,[9 14] ADHD/ADD,[9 17] and depression,[9 14 16] may negatively affect performance in school. These factors are also overrepresented in the paediatric population with obesity. In this study, we can confirm that low parental SES, neuropsychiatric disorders, and anxiety/depression are more common in children with obesity and contribute to the decreased odds of completing ≥12 school years. However, we found that obesity in childhood is a considerable risk factor for not completing ≥12 years of schooling even after taking these and other important risk factors into account.

Obesity treatment outcome and educational level

For peer review only - http://bmjopen.bmj.com/site/about/guidelines.xhtml

We identified a positive association between weight-loss during obesity treatment and completed educational level, which confirms our previously reported data unadjusted for SES.[9] Children with good treatment response, compared with those with no response, were more likely to complete ≥12 school years in all SES groups. A decrease of 0.25 BMI SDS, that has previously been shown to improve metabolic health,[27] we can now also show may have a positive association on completing ≥12 years of schooling. However, the positive effects of successful obesity treatment did not compensate for the observed SES differences on attained level of education.

In Sweden, 12 years of schooling is not mandatory, but the long-term social effects are considerable for those who fail. It has been estimated that 50% of those who fail to complete ≥12 school years face a situation of being left outside the society with poor psychosocial health and large costs for the society.[28] Thus, it is possible that school failure will further worsen the health perspective of adolescents with obesity.

Potential mechanisms between obesity, weight loss, and school performance

Several parameters may interplay when measuring school completion, e.g. resourcefulness, intelligence, or ability to conform. Nevertheless, the mechanisms by which obesity influences school performance are unclear but are likely multifactorial and complex. Possible mechanisms may include psychosocial aspects such as stigma[29] and increased risk for anxiety and depression.[7] Physiological mechanisms may be mediated by anatomical changes such as atrophy of the frontal lobe and reduced hippocampal volume have been demonstrated in individuals with obesity.[30] Some of the reported cognitive dysfunctions in children and adolescents with obesity include slower response times when performing visuospatial attention tasks,[31] diminished executive functions, such as working memory,[32] and slower cognitive

For peer review only - http://bmjopen.bmj.com/site/about/guidelines.xhtml

performance speed.[33] In addition, obesity-related comorbidities such as insulin resistance,[34] type 2 diabetes mellitus,[35] chronic low-grade inflammation,[36] and the metabolic syndrome[37] have also been associated with impaired executive function, memory performance, attention, and cognitive flexibility. A high fat diet in rodents demonstrated detrimental effects on memory and executive functions.[30]

At least some of these effects seem to be reversible, which may add to the positive associations we observed of weight loss on school outcome. Weight loss may have a direct positive effect on cognitive functions.[38] Extensive weight loss via bariatric surgery improves insulin sensitivity and decreases systematic inflammation,[39] and it has been suggested that these factors affect cognitive functions.[40]

However, children with obesity are often stigmatized,[41] have a low self-esteem, and are exposed to bullying and social exclusion.[42] All these factors have most likely a negative impact on school performance. In contrast, a strong social network is most probably an important factor both for good treatment response and achievement in school. An inverse association has been observed between familial social support and child weight status.[43] The relationship between BMI and school completion may also be biased from e.g. assortative mating and dynastic effects which have shown to reduce causal effects.[44] There is also a suggested interplay between genetic variants and environmental factors that may affect intelligence.[45] Thus, it is likely that both negative social effects of obesity and obesity-related morbidity, as well as genetic factors, contribute to the adverse association of childhood obesity on completed educational level.[7 29 41 45]

Limitations

Using longitudinal data from several national registers provided an opportunity to assess the impact of obesity on completed educational level, adjusted for several important confounders.

For peer review only - http://bmjopen.bmj.com/site/about/guidelines.xhtml

Data on both exposure and outcome were prospectively collected and defined according to standardized international classifications.[19 26]

However, some important limitations should be recognized. We did not have anthropometric data on children in the comparison group. There is no representative national data on children with obesity in Sweden. As our comparison group includes children with obesity, although likely less than 1% according to obesity diagnoses found in the National Patient Register, odds of lower level of education associated with obesity might be underestimated. It is also important to consider that children receiving obesity treatment may not be representative of all children with obesity. It should further be noted that parental SES was based on data from one specific year in the child's life and not over the child's entire adolescent lifetime, and that the SES indicators used may not reflect the whole SES spectrum. In addition, the impact of anxiety and depression on educational level may be underestimated since these conditions often are under-diagnosed. We urge the reader to bear in mind that despite several possible mechanisms have been proposed, causal relationships of obesity and the effect of treatment on the outcome remains to be established. Lastly, factors such as free education, school lunches, and students' health care may have an impact on the generalizability of our data to other populations.

Conclusion

In this longitudinal, population-based study, individuals with obesity in childhood were less likely to complete 12 or more years of schooling, compared with a group from the general population. The odds associated with obesity remained significantly increased even after taking parental SES and other important risk factors, such as ADHD, into consideration. The underlying mechanisms are unclear but previous studies indicate that the effects of obesity on school completion both involve psychosocial effects and cognitive functions.[16 17 32] The negative impact of childhood obesity on educational level may partly be reversed by successful

For peer review only

For peer review only - http://bmjopen.bmj.com/site/about/guidelines.xhtml

obesity treatment in childhood. Results from this study underline effects of childhood obesity on schooling. For the sake of an optimal educational environment, it is of great importance to increase awareness both in schools and among decision makers to allocate resources for potential extra support, e.g. reduce stigma and increase educational support.

Acknowledgements

The authors would like to thank all local health care professionals in Sweden working with children and adolescents with obesity and their valuable work with BORIS, and the BORIS steering committee for establishing and maintaining the register.

Author contributions

Conceptualization: Louise Lindberg, Pernilla Danielsson, Martina Persson, Claude Marcus, Emilia Hagman.

Data curation: Louise Lindberg.

Formal analysis: Louise Lindberg, Emilia Hagman.

Funding acquisition: Louise Lindberg, Claude Marcus, Emilia Hagman.

Methodology: Louise Lindberg, Emilia Hagman.

Project administration: Louise Lindberg, Emilia Hagman.

Supervision: Martina Persson, Emilia Hagman, Pernilla Danielsson, Claude Marcus.

Writing – original draft: Louise Lindberg.

Writing – review & editing: Louise Lindberg, Pernilla Danielsson, Martina Persson, Claude Marcus, Emilia Hagman.

Funding statement

This study was supported by funds to LL by Crown Princess Lovisa’s Foundation (2017-00348), Samariten Foundation (2017-0305), the Stockholm FreeMason Foundation for Children’s Welfare, Sällskapet Barnavård, Anna-Lisa och Arne Gustafssons Foundation,

For peer review only

Solstickan Foundation and Sven Jerring Foundation. EH was supported by the Swedish Society for Medical Research, Fredrik and Ingrid Thuring's Foundation (2017-00309) and Magnus Bergvall Foundation (2017-02113), and CM by the Swedish Heart and Lung Foundation (20150790).

Data Sharing statement

The data that support the findings of this study contains sensitive information. Restrictions therefore apply to the availability of these data, which were used under license for the current study, and so are not publicly available. According to Swedish law and the General Data Protection Regulation, the authors are not permitted to share the datasets used in this study with third parties. Given that an ethical approval is obtained, any individual may apply for data from Statistics Sweden via information@scb.se, the Swedish National Board of Health and Welfare via registerservice@socialstyrelsen.se, and the Swedish Childhood Obesity Treatment Register via http://www.e-boris.se/kontaktuppgifter/.

Competing interests

All authors have completed the ICMJE uniform disclosure form at www.icmje.org/coi_disclosure.pdf and declare: no support from any organisation for the submitted work; no financial relationships with any organisations that might have an interest in the submitted work in the previous three years; no other relationships or activities that could appear to have influenced the submitted.

For peer review only - http://bmjopen.bmj.com/site/about/guidelines.xhtml

References

1. Abarca-Gómez L, Abdeen ZA, Hamid ZA, et al. Worldwide trends in body-mass index, underweight, overweight, and obesity from 1975 to 2016: a pooled analysis of 2416 population-based measurement studies in 128.9 million children, adolescents, and adults. *The Lancet* 2017 doi: 10.1016/S0140-6736(17)32129-3

2. Hagman E, Danielsson P, Brandt L, et al. Association between impaired fasting glycaemia in pediatric obesity and type 2 diabetes in young adulthood. *Nutr Diabetes* 2016;6(8):e227. doi: 10.1038/nutd.2016.34 [published Online First: 2016/08/23]

3. Hagman E, Danielsson P, Elimam A, et al. The effect of weight loss and weight gain on blood pressure in children and adolescents with obesity. *Int J Obes (Lond)* 2019;43(10):1988-94. doi: 10.1038/s41366-019-0384-2 [published Online First: 2019/06/04]

4. Lindberg L, Danielsson P, Persson M, et al. Association of childhood obesity with risk of early all-cause and cause-specific mortality: A Swedish prospective cohort study. *PLoS medicine* 2020;17(3):e1003078. doi: 10.1371/journal.pmed.1003078 [published Online First: 2020/03/19]

5. Reilly JJ, Kelly J. Long-term impact of overweight and obesity in childhood and adolescence on morbidity and premature mortality in adulthood: systematic review. *Int J Obes (Lond)* 2011;35(7):891-8. doi: 10.1038/ijo.2010.222

6. Viner RM, Cole TJ. Adult socioeconomic, educational, social, and psychological outcomes of childhood obesity: a national birth cohort study. *BMJ* 2005;330(7504):1354. doi: 10.1136/bmj.38453.422049.E0 [published Online First: 2005/05/20]

7. Lindberg L, Hagman E, Danielsson P, et al. Anxiety and depression in children and adolescents with obesity: a nationwide study in Sweden. *BMC medicine* 2020;18(1):30. doi: 10.1186/s12916-020-1498-z [published Online First: 2020/02/23]

8. Laitinen J, Power C, Ek E, et al. Unemployment and obesity among young adults in a northern Finland 1966 birth cohort. *International journal of obesity and related metabolic disorders : journal of the International Association for the Study of Obesity* 2002;26(10):1329-38. doi: 10.1038/sj.ijo.0802134

9. Hagman E, Danielsson P, Brandt L, et al. Childhood Obesity, Obesity Treatment Outcome, and Achieved Education: A Prospective Cohort Study. *The Journal of adolescent health : official publication of the Society for Adolescent Medicine* 2017 doi: 10.1016/j.jadohealth.2017.04.009 [published Online First: 2017/07/12]

10. Karnehed N, Rasmussen F, Hemmingsson T, et al. Obesity and attained education: cohort study of more than 700,000 Swedish men. *Obesity* 2006;14(8):1421-28.

11. French SA, Wall M, Corbeil T, et al. Obesity in Adolescence Predicts Lower Educational Attainment and Income in Adulthood: The Project EAT Longitudinal Study. *Obesity (Silver Spring)* 2018;26(9):1467-73. doi: 10.1002/oby.22273 [published Online First: 2018/09/19]

12. Shrewsbury V, Wardle J. Socioeconomic status and adiposity in childhood: a systematic review of cross-sectional studies 1990-2005. *Obesity (Silver Spring)* 2008;16(2):275-84. doi: 10.1038/oby.2007.35 [published Online First: 2008/02/02]

13. Koivusilta LK, West P, Saaristo VM, et al. From childhood socio-economic position to adult educational level - do health behaviours in adolescence matter? A longitudinal study. *BMC public health* 2013;13:711. doi: 10.1186/1471-2458-13-711

14. Kark M, Hjern A, Rasmussen F. Poor school performance is associated with a larger gain in body mass index during puberty. *Acta Paediatr* 2014;103(2):207-13. doi: 10.1111/apa.12471

15. DeGarmo DS, Forgatch MS, Martinez CR, Jr. Parenting of divorced mothers as a link between social status and boys' academic outcomes: unpacking the effects of socioeconomic status. *Child development* 1999;70(5):1231-45. [published Online First: 1999/11/05]

For peer review only

For peer review only - http://bmjopen.bmj.com/site/about/guidelines.xhtml

16. Freeman A, Tyrovolas S, Koyanagi A, et al. The role of socio-economic status in depression: results from the COURAGE (aging survey in Europe). *BMC public health* 2016;16(1):1098. doi: 10.1186/s12889-016-3638-0 [published Online First: 2016/10/21]

17. Scholtens S, Rydell AM, Yang-Wallentin F. ADHD symptoms, academic achievement, self-perception of academic competence and future orientation: a longitudinal study. *Scandinavian journal of psychology* 2013;54(3):205-12. doi: 10.1111/sjop.12042 [published Online First: 2013/03/21]

18. Quek YH, Tam WWS, Zhang MWB, et al. Exploring the association between childhood and adolescent obesity and depression: a meta-analysis. *Obesity reviews : an official journal of the International Association for the Study of Obesity* 2017;18(7):742-54. doi: 10.1111/obr.12535 [published Online First: 2017/04/13]

19. Cole TJ, Lobstein T. Extended international (IOTF) body mass index cut-offs for thinness, overweight and obesity. *Pediatr Obes* 2012;7(4):284-94. doi: 10.1111/j.2047-6310.2012.00064.x

20. Hagman E, Danielsson P, Lindberg L, et al. Paediatric obesity treatment during 14 years in Sweden: Lessons from the Swedish Childhood Obesity Treatment Register-BORIS. *Pediatr Obes* 2020;15(7):e12626. doi: 10.1111/ijpo.12626 [published Online First: 2020/02/20]

21. Ludvigsson JF, Otterblad-Olausson P, Pettersson BU, et al. The Swedish personal identity number: possibilities and pitfalls in healthcare and medical research. *European journal of epidemiology* 2009;24(11):659-67. doi: 10.1007/s10654-009-9350-y

22. Ludvigsson JF, Almqvist C, Bonamy AK, et al. Registers of the Swedish total population and their use in medical research. *European journal of epidemiology* 2016;31(2):125-36. doi: 10.1007/s10654-016-0117-y [published Online First: 2016/01/16]

23. Ludvigsson JF, Andersson E, Ekbom A, et al. External review and validation of the Swedish national inpatient register. *BMC public health* 2011;11:450. doi: 10.1186/1471-2458-11-450 [published Online First: 2011/06/11]

24. Wallerstedt SM, Wettermark B, Hoffmann M. The First Decade with the Swedish Prescribed Drug Register - A Systematic Review of the Output in the Scientific Literature. *Basic & clinical pharmacology & toxicology* 2016;119(5):464-69. doi: 10.1111/bcpt.12613 [published Online First: 2016/04/27]

25. Brooke HL, Talback M, Hornblad J, et al. The Swedish cause of death register. *European journal of epidemiology* 2017;32(9):765-73. doi: 10.1007/s10654-017-0316-1 [published Online First: 2017/10/07]

26. UNESCO. International standard classification of education: Isced 1997. Re-edition. Paris: UNESCO Institute for Statistics. 2011

27. Ford AL, Hunt LP, Cooper A, et al. What reduction in BMI SDS is required in obese adolescents to improve body composition and cardiometabolic health? *Archives of disease in childhood* 2010;95(4):256-61. doi: 10.1136/adc.2009.165340 [published Online First: 2009/12/08]

28. Nilsson I, Wadeskog A. Utanförskapets Ekonomiska Sociotoper: Socioekonomisk analys på stadsdelsnivå inom ramen för Healthy Cities 2012

29. Finn KE, Seymour CM, Phillips AE. Weight bias and grading among middle and high school teachers. *Br J Educ Psychol* 2019 doi: 10.1111/bjep.12322 [published Online First: 2019/10/28]

30. Nguyen JC, Killcross AS, Jenkins TA. Obesity and cognitive decline: role of inflammation and vascular changes. *Frontiers in neuroscience* 2014;8:375. doi: 10.3389/fnins.2014.00375 [published Online First: 2014/12/06]

31. Tsai C-L, Chen F-C, Pan C-Y, et al. The neurocognitive performance of visuospatial attention in children with obesity. *Frontiers in psychology* 2016;7

32. Laurent JS, Watts R, Adise S, et al. Associations Among Body Mass Index, Cortical Thickness, and Executive Function in Children. *JAMA Pediatr* 2019 doi: 10.1001/jamapediatrics.2019.4708 [published Online First: 2019/12/10]

For peer review only

For peer review only - http://bmjopen.bmj.com/site/about/guidelines.xhtml

33. Sweat V, Yates KF, Migliaccio R, et al. Obese adolescents show reduced cognitive processing speed compared with healthy weight peers. *Childhood Obesity* 2017

34. Cheke LG, Bonnici HM, Clayton NS, et al. Obesity and insulin resistance are associated with reduced activity in core memory regions of the brain. *Neuropsychologia* 2017;96:137-49.

35. Bruehl H, Sweat V, Tirsi A, et al. Obese Adolescents with Type 2 Diabetes Mellitus Have Hippocampal and Frontal Lobe Volume Reductions. *Neuroscience and medicine* 2011;2(1):34-42. doi: 10.4236/nm.2011.21005

36. Spyridaki EC, Simos P, Avgoustinaki PD, et al. The association between obesity and fluid intelligence impairment is mediated by chronic low-grade inflammation. *British Journal of Nutrition* 2014;112(10):1724-34.

37. Mangone A, Yates KF, Sweat V, et al. Cognitive functions among predominantly minority urban adolescents with metabolic syndrome. *Applied Neuropsychology: Child* 2017:1-7.

38. Alosco ML, Spitznagel MB, Strain G, et al. Improved memory function two years after bariatric surgery. *Obesity* 2014;22(1):32-38.

39. Olbers T, Beamish AJ, Gronowitz E, et al. Laparoscopic Roux-en-Y gastric bypass in adolescents with severe obesity (AMOS): a prospective, 5-year, Swedish nationwide study. *Lancet Diabetes Endocrinol* 2017;5(3):174-83. doi: 10.1016/S2213-8587(16)30424-7

40. Biessels GJ, Reagan LP. Hippocampal insulin resistance and cognitive dysfunction. *Nature Reviews Neuroscience* 2015;16(11):660.

41. Latner JD, Stunkard AJ. Getting worse: the stigmatization of obese children. *Obesity research* 2003;11(3):452-6. doi: 10.1038/oby.2003.61

42. Pierce JW, Wardle J. Cause and effect beliefs and self-esteem of overweight children. *Journal of child psychology and psychiatry, and allied disciplines* 1997;38(6):645-50. [published Online First: 1997/10/08]

43. Lindberg L, Ek A, Nyman J, et al. Low grandparental social support combined with low parental socioeconomic status is closely associated with obesity in preschool-aged children: a pilot study. *Pediatr Obes* 2015 doi: 10.1111/ijpo.12049

44. Brumpton B, Sanderson E, Heilbron K, et al. Avoiding dynastic, assortative mating, and population stratification biases in Mendelian randomization through within-family analyses. *Nature communications* 2020;11(1):3519. doi: 10.1038/s41467-020-17117-4 [published Online First: 2020/07/16]

45. Plomin R, von Stumm S. The new genetics of intelligence. *Nature reviews Genetics* 2018;19(3):148-59. doi: 10.1038/nrg.2017.104 [published Online First: 2018/01/18]

For peer review only

For peer review only - http://bmjopen.bmj.com/site/about/guidelines.xhtml

Supporting information

S1 Table. International Classification of Diseases (ICD 10th revision) codes and Anatomical Therapeutic Chemical (ATC) classification system codes used.

S2 Table. Proportion of children completing ≥12 years of schooling.

S3 Table. Proportion of individuals who have undergone obesity treatment in childhood who completed ≥12 years of schooling by calendar year and by age at start of obesity treatment (n=3,942).

Figure Legends

Fig 1. Participant flowchart.

Fig 2. Percentage of individuals completing ≥12 years of schooling by SES level in the childhood obesity cohort (n=3,921,) and the comparison group (n=18,528).

Fig 3. Crude percentage of individuals with obesity in childhood (n=3,921) completing ≥12 school years by parental SES and treatment response. p=p for trend; n=refers to number of individuals in each category.

For peer review only - http://bmjopen.bmj.com/site/about/guidelines.xhtml

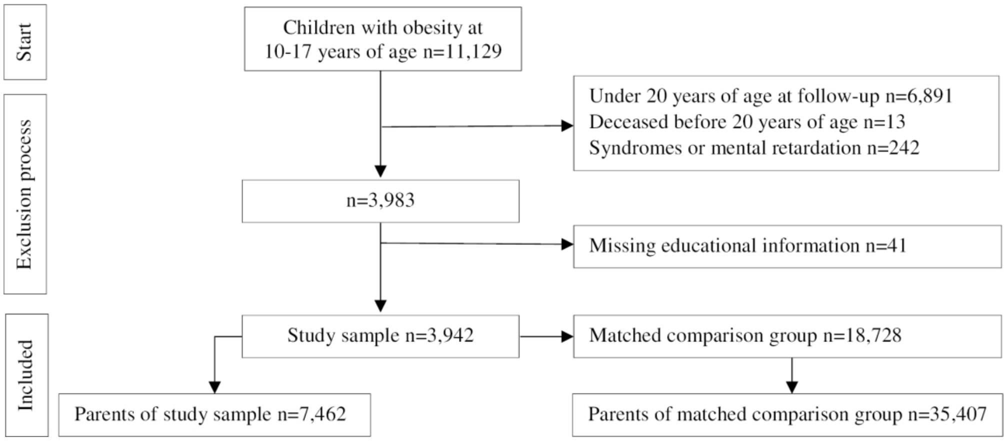

Figure 1

169x73mm (300 x 300 DPI)

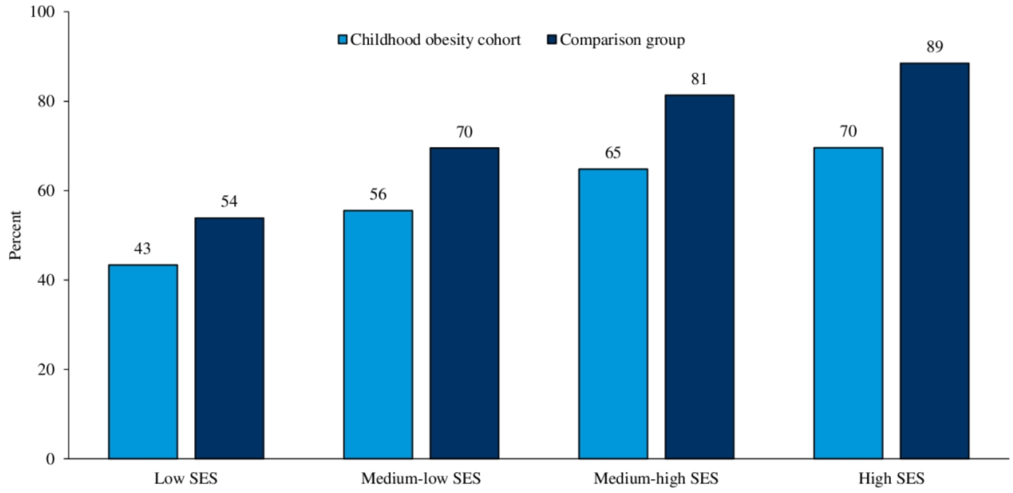

Fig 2. Percentage of individuals completing ≥12 years of schooling by SES level in the childhood obesity cohort (n=3,921) and the comparison group (n=18,528).

118x57mm (300 x 300 DPI)

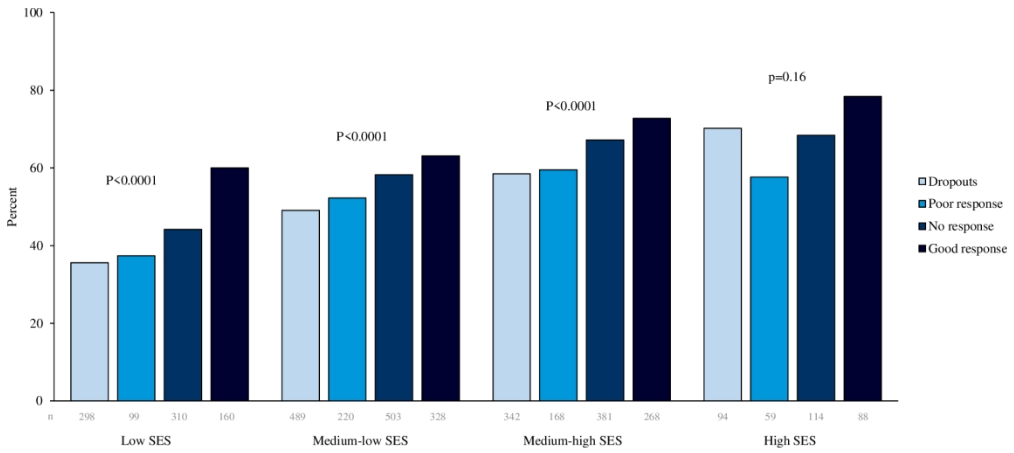

Fig 3. Crude percentage of individuals with obesity in childhood (n=3,921) completing ≥12 school years by parental SES and treatment response. p=p for trend; n=refers to number of individuals in each category.

150x66mm (300 x 300 DPI)

S1 Table. International Classification of Diseases (ICD 10th revision) codes and Anatomical Therapeutic Chemical (ATC) classification system codes used.

Condition	ICD-10	ATC Code
Anxiety/sedatives	F40.0-F40.1, F41-F42, F51.4-F51.9, F93.0-F93.2	N05B
Depression/antidepressants	F32-F39, F92	N06A
ADHD/ADD	F90	N06B
Mental retardation	F70-F79	
Genetic syndromes		
Fragile X	Q99.2	
Klinefelter	Q98	
Laurence-Moon-Bardet-Biedl	Q87.8B	
Mb Down	Q90	
Noonan	Q87.1E	
Prader-Willi	Q87.1F	
Silver Russell	Q87.1G	
Turner	Q96	

For peer review only

S2 Table. Proportion of children completing ≥12 years of schooling

	Childhood obesity cohort		Comparison group		
	n	%	n	%	p-value¶
Total	2,236	56.7	13,939	74.4	<0.0001
Sex					
Girl	1,090	59.7	6,843	78.7	<0.0001
Boy	1,146	54.1	7,096	70.8	<0.0001
Migration background					
Nordic	1,695	58.4	10,974	78.1	<0.0001
Non-Nordic	541	52.2	2,965	63.4	<0.0001
ADHD/ADD					
Non-ADHD/ADD	2,032	61.1	13,528	76.5	<0.0001
ADHD/ADD	204	33.1	411	39.4	0.01
Anxiety/depression					
No anxiety/depression	1,902	61.1	12,754	77.0	<0.0001
Anxiety/depression	334	40.2	1,185	54.9	<0.0001
Parental SES					
Low	376	43.4	1,455	53.9	<0.0001
Medium-low	855	55.5	4,187	69.5	<0.0001
Medium-high	751	64.8	5,358	81.3	<0.0001
High	247	69.6	2,848	88.5	<0.0001
Maternal education					
Compulsory school	755	47.7	3,474	61.9	<0.0001
Upper secondary school	945	61.3	5,582	77.9	<0.0001
University degree	506	69.3	4,589	85.3	<0.0001
Paternal education					
Compulsory school	639	50.2	2,775	63.0	<0.0001
Upper secondary school	1,132	59.9	7,085	77.9	<0.0001
University degree	318	66.0	3,333	85.2	<0.0001
Maternal income					
Q1	614	52.3	3,327	68.1	<0.0001
Q2	750	58.6	4,391	75.2	<0.0001
Q3	542	58.2	3,534	77.1	<0.0001
Q4	304	62.6	2,429	81.3	<0.0001
Paternal income					
Q1	556	49.3	2,453	62.6	<0.0001
Q2	410	54.0	2,260	72.3	<0.0001
Q3	549	61.8	3,404	77.6	<0.0001
Q4	572	66.2	5,056	84.5	<0.0001
Maternal occupational status					
No occupation	438	47.8	1,722	58.3	<0.0001
Occupation	1,777	60.0	12,005	77.9	<0.0001
Paternal occupational status					
No occupation	363	45.4	1,543	58.7	<0.0001
Occupation	1,736	60.5	11,703	78.5	<0.0001

Abbreviations: SES, socioeconomic status; Q, quartile. ¶Performed with chi-square.
1 EUR ≈ 9.83 SEK December 31st 2017. Q1: <18,652 EUR; Q2: 18,652 to 25,025 EUR; Q3: 25,026 to 32,309 EUR; Q4: >32,309 EUR.

For peer review only

S3 Table. Proportion of individuals who have undergone obesity treatment in childhood who completed ≥12 years of schooling by calendar year and by age at start of obesity treatment (n=3,942).

Individuals completing ≥12 years of schooling		
	n	%
Calendar year at start of obesity treatment		
1995-2001	229	62.6
2002-2008	1,262	58.1
2009-2014	745	53.1
Age at start of obesity treatment		
10 - 12.99	960	59.0
13 - 16.99	1,276	55.1

For peer review only

STROBE Statement—Checklist of items that should be included in reports of ***cohort studies***

	Item No	Recommendation	Page No
**Title and abstract**	1	(*a*) Indicate the study's design with a commonly used term in the title or the abstract	1-2
		(*b*) Provide in the abstract an informative and balanced summary of what was done and what was found	2-3
**Introduction**			
Background/rationale	2	Explain the scientific background and rationale for the investigation being reported	4
Objectives	3	State specific objectives, including any prespecified hypotheses	4.5
**Methods**			
Study design	4	Present key elements of study design early in the paper	5
Setting	5	Describe the setting, locations, and relevant dates, including periods of recruitment, exposure, follow-up, and data collection	5-9
Participants	6	(*a*) Give the eligibility criteria, and the sources and methods of selection of participants. Describe methods of follow-up	5
		(*b*) For matched studies, give matching criteria and number of exposed and unexposed	5
Variables	7	Clearly define all outcomes, exposures, predictors, potential confounders, and effect modifiers. Give diagnostic criteria, if applicable	7-9
Data sources/ measurement	8*	For each variable of interest, give sources of data and details of methods of assessment (measurement). Describe comparability of assessment methods if there is more than one group	7-9
Bias	9	Describe any efforts to address potential sources of bias	7-9
Study size	10	Explain how the study size was arrived at	5
Quantitative variables	11	Explain how quantitative variables were handled in the analyses. If applicable, describe which groupings were chosen and why	9-10
Statistical methods	12	(*a*) Describe all statistical methods, including those used to control for confounding	9-10
		(*b*) Describe any methods used to examine subgroups and interactions	
		(*c*) Explain how missing data were addressed	
		(*d*) If applicable, explain how loss to follow-up was addressed	
		(*e*) Describe any sensitivity analyses	
**Results**			
Participants	13*	(a) Report numbers of individuals at each stage of study—eg numbers potentially eligible, examined for eligibility, confirmed eligible, included in the study, completing follow-up, and analysed	12 + fig
		(b) Give reasons for non-participation at each stage	
		(c) Consider use of a flow diagram	
Descriptive data	14*	(a) Give characteristics of study participants (eg demographic, clinical, social) and information on exposures and potential confounders	11
		(b) Indicate number of participants with missing data for each variable of interest	
		(c) Summarise follow-up time (eg, average and total amount)	
Outcome data	15*	Report numbers of outcome events or summary measures over time	12

For peer review only - http://bmjopen.bmj.com/site/about/guidelines.xhtml

Main results	16	(*a*) Give unadjusted estimates and, if applicable, confounder-adjusted estimates and their precision (eg, 95% confidence interval). Make clear which confounders were adjusted for and why they were included<br>(*b*) Report category boundaries when continuous variables were categorized<br>(*c*) If relevant, consider translating estimates of relative risk into absolute risk for a meaningful time period	12-17
Other analyses	17	Report other analyses done—eg analyses of subgroups and interactions, and sensitivity analyses	12-17
**Discussion**			
Key results	18	Summarise key results with reference to study objectives	18
Limitations	19	Discuss limitations of the study, taking into account sources of potential bias or imprecision. Discuss both direction and magnitude of any potential bias	20-21
Interpretation	20	Give a cautious overall interpretation of results considering objectives, limitations, multiplicity of analyses, results from similar studies, and other relevant evidence	18-21
Generalisability	21	Discuss the generalisability (external validity) of the study results	21
**Other information**			
Funding	22	Give the source of funding and the role of the funders for the present study and, if applicable, for the original study on which the present article is based	22-23

*Give information separately for exposed and unexposed groups.

Note: An Explanation and Elaboration article discusses each checklist item and gives methodological background and published examples of transparent reporting. The STROBE checklist is best used in conjunction with this article (freely available on the Web sites of PLoS Medicine at http://www.plosmedicine.org/, Annals of Internal Medicine at http://www.annals.org/, and Epidemiology at http://www.epidem.com/). Information on the STROBE Initiative is available at http://www.strobe-statement.org.

For peer review only

BMJ Open

Obesity in childhood, socioeconomic status, and completion of 12 or more school years: a prospective cohort study

Journal:	*BMJ Open*
Manuscript ID	bmjopen-2020-040432.R2
Article Type:	Original research
Date Submitted by the Author:	02-Dec-2020

Complete List of Authors:	Lindberg, Louise; Karolinska Institutet, Dept. Clinical Science, Intervention and Technology<br>Persson, Martina; Karolinska Institute, Department of Medicine, Clinical Epidemiology, Karolinska Institutet;<br>Danielsson, Pernilla; Karolinska Institutet, Dept. Clinical Science, Intervention and Technology<br>Hagman, Emilia; Karolinska Institutet, Dept. Clinical Science, Intervention and Technology<br>Marcus, Claude; Karolinska Institutet, Dept. Clinical Science, Intervention and Technology

**Primary Subject Heading**:	Paediatrics
Secondary Subject Heading:	Epidemiology, Nutrition and metabolism, Public health
Keywords:	Paediatric endocrinology < DIABETES & ENDOCRINOLOGY, EPIDEMIOLOGY, Paediatric endocrinology < PAEDIATRICS, PUBLIC HEALTH

SCHOLARONE™
Manuscripts

I, the Submitting Author has the right to grant and does grant on behalf of all authors of the Work (as defined in the below author licence), an exclusive licence and/or a non-exclusive licence for contributions from authors who are: i) UK Crown employees; ii) where BMJ has agreed a CC-BY licence shall apply, and/or iii) in accordance with the terms applicable for US Federal Government officers or employees acting as part of their official duties; on a worldwide, perpetual, irrevocable, royalty-free basis to BMJ Publishing Group Ltd ("BMJ") its licensees and where the relevant Journal is co-owned by BMJ to the co-owners of the Journal, to publish the Work in this journal and any other BMJ products and to exploit all rights, as set out in our licence.

The Submitting Author accepts and understands that any supply made under these terms is made by BMJ to the Submitting Author unless you are acting as an employee on behalf of your employer or a postgraduate student of an affiliated institution which is paying any applicable article publishing charge ("APC") for Open Access articles. Where the Submitting Author wishes to make the Work available on an Open Access basis (and intends to pay the relevant APC), the terms of reuse of such Open Access shall be governed by a Creative Commons licence – details of these licences and which Creative Commons licence will apply to this Work are set out in our licence referred to above.

Other than as permitted in any relevant BMJ Author's Self Archiving Policies, I confirm this Work has not been accepted for publication elsewhere, is not being considered for publication elsewhere and does not duplicate material already published. I confirm all authors consent to publication of this Work and authorise the granting of this licence.

For peer review only

Obesity in childhood, socioeconomic status, and completion of 12 or more school years: a prospective cohort study

Louise Lindberg[1], Martina Persson[2,3,4], Pernilla Danielsson[1], Emilia Hagman[1*], Claude Marcus[1]

[1]Department of Clinical Science, Intervention and Technology, Division of Pediatrics, Karolinska Institutet, Stockholm, Sweden

[2]Department of Medicine Solna, Clinical Epidemiology Unit, Karolinska Institutet, Stockholm, Sweden

[3]Department of Diabetes and Endocrinology, Sachsska Children's Hospital Södersjukhuset, Stockholm, Sweden

[4]Department of Clinical Science and Education, Södersjukhuset, Karolinska Institutet, Stockholm, Sweden

*Corresponding author e-mail: emilia.hagman @ki.se (EH)

Other contributing authors e-mail: louise.lindberg@ki.se; martina.persson@ki.se; pernilla.danielsson.liljeqvist@ki.se; claude.marcus@ki.se

Keywords: Pediatric obesity, schools, epidemiology, social class, cohort study

Word count: 3963

For peer review only - http://bmjopen.bmj.com/site/about/guidelines.xhtml

Abstract

Objectives: Children with obesity achieve lower educational level compared with normal-weight peers. Parental socioeconomic status (SES) impacts both a child's academic achievement and risk of obesity. The degree to which the association between obesity and education depend on parental SES is unclear. Therefore, the primary aim is to investigate if individuals with obesity in childhood are less likely to complete ≥12 years of schooling, independently of parental SES. The secondary aim is to study how weight loss, level of education, and parental SES are associated.

Design: Nationwide prospective cohort study.

Setting: Swedish national register data.

Participants: Children aged 10-17 years, recorded in the Swedish Childhood Obesity Treatment Register (BORIS), and aged 20 years or older at follow-up were included (n=3,942). A comparison group was matched by sex, year of birth, and living area (n=18,728). Parental SES was based on maternal and paternal level of education, income, and occupational status.

Primary outcome measure: Completion of ≥12 years of schooling was analysed with conditional logistic regression, and adjusted for group, migration background, ADHD/ADD, anxiety/depression, and parental SES.

Results: Among those with obesity in childhood, 56.7% completed ≥12 school years compared with 74.4% in the comparison group ($p<0.0001$). High parental SES compared with low SES was strongly associated with attained level of education in both children with and without obesity, adjusted odds ratio (^{a}OR) [99% confidence interval (CI)]=5.40 [4.45 to 6.55]. However, obesity in childhood remains a strong risk factor of not completing ≥12

For peer review only - http://bmjopen.bmj.com/site/about/guidelines.xhtml

school years, independently of parental SES, [a]OR=0.57 [0.51 to 0.63]. Successful obesity treatment increased the odds of completing ≥12 years in school even when taking parental SES into account, [a]OR=1.34 [1.04 to 1.72].

Conclusions: Individuals with obesity in childhood have lower odds of completing ≥12 school years, independently of parental SES. Optimized obesity treatment may improve school results in this group.

Strengths and limitation of this study

- In this prospective cohort study, we have been able to investigate the level of education among a large number of individuals who have obesity in childhood (n= 3,942) in comparison with a matched group (n=18,728).
- The study design of using longitudinal data from several national registers provided the opportunity to control for important confounding factors, such as neuropsychiatric disorders, anxiety, depression, and family socioeconomic status.
- Factors such as free education, school lunches, and students' health care may have an impact on the generalizability of our data to other populations.

For peer review only - http://bmjopen.bmj.com/site/about/guidelines.xhtml

Introduction

During the last 40 years, the prevalence of childhood obesity has increased exponentially in many parts of the world.[1] Childhood obesity is associated with increased risks of somatic morbidity and risk of premature death in adulthood.[2-5] Psychosocial maladjustment, anxiety and depression are also prevalent[6 7] and may contribute to the obesity-related long-term morbidity and mortality.[5] Most studies report that children with obesity more often have lower school grades and reach a lower level of education compared with normal-weight peers,[8-10] but in a recent study, only girls with obesity were affected.[11] We have previously confirmed the association between obesity and lower attained level of education among both girls and boys.[9] We also found that successful obesity treatment was positively correlated with completing ≥12 school years, although without taking socioeconomic status (SES) into account.[9]

Low parental SES is a well-established risk factor for both childhood obesity and poorer academic achievement.[12] In particular, parental education has been demonstrated to influence the child's performance at school.[13-15] In addition, depression[16] and neuropsychiatric disorders, including Attention Deficit Disorder with or without Hyperactivity (ADHD/ADD),[17] may adversely affect school results. Depression and neuropsychiatric disorders are more common in children with obesity compared to the general population.[9 18] Investigating the impact of obesity alone on attained level of education requires that these and other confounders are considered. The primary aim of this study was to disentangle the association of childhood obesity and parental SES on completed level of education. The

For peer review only - http://bmjopen.bmj.com/site/about/guidelines.xhtml

secondary aim was to study if positive effects of weight loss on attained level of education is affected by parental SES.

Methods

Study population

This prospective cohort study included children with obesity,[19] aged 10-17 years at the start of obesity treatment (December 1994 to December 2015), and aged 20 years or older and living in Sweden at follow-up (December 31st 2017; n=3,942). Data on subjects receiving treatment for childhood obesity was collected from the Swedish Childhood Obesity Treatment Register (BORIS). BORIS has been thoroughly described elsewhere,[20] but in short: The main purpose of the register is quality assessment and long-term evaluation of childhood obesity treatment. Treatment is individualized and consists primarily of lifestyle modification (i.e. diet and physical activity).

A comparison group from the general population was randomly identified using the Swedish Total Population Register and matched by sex, year of birth, and living area at the year obesity treatment was initiated (n=18,728). Using density matching without replacement, five individuals were matched to each individual from the childhood obesity cohort. Siblings of children registered in the childhood obesity cohort were excluded from the comparison group. Children with a diagnosis of mental retardation or genetic syndromes were excluded from both the childhood obesity cohort and the comparison group (Fig 1 and S1 Table).

For peer review only - http://bmjopen.bmj.com/site/about/guidelines.xhtml

Families were informed in written or verbal about data collection in the Swedish Childhood Obesity Treatment Register. Post an opt-out approval by parents/guardians, data of the children's weight and height were recorded by the local health care provider during treatment visits. There was no data of weight and height of individuals in the comparison group. However, less than 1% of the individuals in the comparison group were found in the National Patient Register with a diagnosis of obesity in childhood. In Sweden, health care is free of charge for children and adolescents until 18 years of age. The study was approved by the regional Ethics Committee in Stockholm, Sweden (No. 2016/922-31/1).

Data sources

Using the Swedish identity number, which is unique to each resident in Sweden, data from several national registers were linked.[21] Data on education, income, and occupational status were obtained from the Longitudinal Integration Database for Health Insurance and Labour Market Studies. Information on migration background for children and their parents was obtained from the Swedish Total Population Register.[22] Both these registers are held by Statistics Sweden, a governmental agency that collects and provide official statistics (www.scb.se/en).

Data on diagnoses of mental retardation, genetic syndromes, ADHD/ADD, anxiety, and depression were identified based on codes according the International Classification of Diseases (10th revision; S1 Table) and retrieved from the National Patient Register.[23] Information on prescriptions of psychostimulant drugs for ADHD/ADD as well as antidepressants and tranquilizers for anxiety and depression were identified using the Anatomical Therapeutic Chemical classification system (S1 Table) retrieved from the

For peer review only - http://bmjopen.bmj.com/site/about/guidelines.xhtml

Swedish Prescribed Drug Register.[24] Data on deaths were retrieved from the Cause of Death Register.[25] These registers are held and were linked by the governmental agency the National Board of Health and Welfare (www.socialstyrelsen.se/english).

Definition of outcome

The main outcome was defined as completion of ≥12 years of schooling, and based on the International Standard Classification of Education.[26] In Sweden, children start school at the age of 6 or 7 years and attend compulsory school for 9 years. Upper secondary school includes three additional years of schooling and provides the requirements to attend higher education. Usually students graduate from upper secondary school at 18 or 19 years of age. All education in Sweden is free of charge. Students in compulsory and upper secondary school are provided school lunches and health care at schools, also free of charge.

Definition of exposure variables

Degree of obesity in children from BORIS was assessed with Body Mass Index Standard Deviation Score (BMI SDS), which is standardized by sex and age and used to measure degree of obesity in growing children.[19] Baseline measures (continuous) were used for the variable BMI SDS at start of treatment. Response to obesity treatment was based on the change of BMI SDS from the first to the last clinical visit and categorized into four groups:[3, 27] good response, a reduction of BMI SDS by 0.25 units or more; no response, a change of BMI SDS by +/- 0.24 units; poor response, an increase of BMI SDS by 0.25 units or more; and dropouts, children with less than one year between their first and last measure or without clinical follow-up after their first registered visit.

For peer review only - http://bmjopen.bmj.com/site/about/guidelines.xhtml

Parental SES was based on maternal and paternal level of education, income, and occupational status at the year the child turned 15 years, which is about the same time as the child starts upper secondary school. In case of the child was adopted, the SES of adoptive parents was used (childhood obesity cohort n=24 and comparison group n=164). The rationale of treating SES as a composite variable was to capture more of the social context and a potential inequality embedded there. Thus, by taking three variables into account instead of one, we get a more wide and robust measure of SES. Maternal and paternal educational level was categorized into compulsory school, upper secondary school, or university degree. Annual disposable income was used to reflect maternal and paternal economic capacity. The annual disposable income includes all taxable (direct labour income, capital gains from shares etc.) and tax-free income (housing and child benefits, student aid etc.), minus final tax and other negative transfers such as capital loss from shares and properties. Disposable income from different years was converted to 2017 prices using the Consumer Price Index for Sweden provided by Statistics Sweden (www.scb.se/en). Income was categorized into quartiles based on data from the parents in the comparison group**.** No occupation was defined as either unemployment 6 months or more, or income from long-term sick leave exceeding any income from the individual's gross salary. Individuals considered to have an occupation included those registered as employed or having an income from student grants/loan equivalent to full-time studies for at least one semester. Parental SES was weighted according to maternal and paternal education (0, 1, 2), income (0, 1, 2, 3), and occupational status (0, 1). The mean parental SES score (i.e. the sum of the mother's and father's all SES indicators divided by two) was applied to their child. The SES-variable was

For peer review only - http://bmjopen.bmj.com/site/about/guidelines.xhtml

categorized into four levels; low SES (0 to 1.5 points), medium-low SES (2 to 3 points), medium-high SES (3.5 to 4.5 points), and high SES (5 to 6 points).

The prevalence of both ADHD/ADD[9] and depression[18] is higher in children with obesity and may negatively influence attained level of education.[9 16 17] ADHD/ADD and anxiety/depression in children were identified based on diagnosis or dispensed prescribed medication (S1 Table).

Definition of covariates

The migration background of children in Sweden may impact school achievements and is therefore an important factor to control for in the analyses.[9 14] Migration background was categorized as Nordic or non-Nordic. Nordic was defined as born in a Nordic country (Sweden, Norway, Denmark, Finland, or Iceland) with one or two parents born in the Nordic. Children were classified as non-Nordic if born outside the Nordic or born in the Nordic with two parents born outside the Nordic. Other covariates included sex and age (continuous) at start of obesity treatment.

Patient and public involvement

No patient involved.

Statistical analysis

Descriptive statistics are presented as means and confidence intervals (CI), medians and interquartile ranges (IQR), or frequencies and percentages. Conditional logistic regression

For peer review only - http://bmjopen.bmj.com/site/about/guidelines.xhtml

186 was used to calculate odds ratios (OR's) with 99% CI for the main outcome, i.e. completing
187 ≥12 school years. Conditional logistic regression was used above ordinary logistic regression
188 as the childhood obesity cohort and the comparison group were matched by several variables.
189 Independent variables included in the adjusted analyses were migration background,
190 ADHD/ADD, anxiety/depression, parental SES, and group (childhood obesity cohort or
191 comparison group) in analyses including both groups, otherwise stratified by group.
192 Interaction between childhood obesity and parental SES was tested. The association of each
193 SES indicator on the odds of completing ≥12 school years was also analysed separately for
194 mothers and fathers. Sensitivity analyses excluding individuals with a non-Swedish
195 background (defined as child born in Sweden with at least one parent also born in Sweden),
196 or ADHD/ADD, or anxiety/depression were performed.

197 Secondary analyses were performed within the childhood obesity cohort to examine
198 associations between patient characteristics and completed educational level. In ordinary
199 logistic regressions, odds were adjusted for sex, migration background, parental SES, age
200 and BMI SDS at start of treatment, treatment response, ADHD/ADD, and
201 anxiety/depression. We tested for possible interaction between parental SES and treatment
202 response for the odds of completing ≥12 years of schooling.

203 As missing data on parental SES was rare in both groups (Table 1), records with missing data
204 were excluded from the analyses, i.e. data were not imputed. P-values <0.01 were considered
205 statistically significant. All analyses were performed using SAS statistical software (version
206 9.4, Cary, NC, USA).

207

For peer review only

For peer review only - http://bmjopen.bmj.com/site/about/guidelines.xhtml

Table 1. Characteristics of the participants (n=22,670)

	Childhood obesity cohort (n=3,942)		Comparison group (n=18,728)	
	n	%	n	%
Female sex	1,825	46.3	8,701	46.5
Nordic	2,905	73.7	14,048	75.0
Age at end of follow-up (years)	23.4*	21.4-26.3*	23.4*	21.5-26.3*
ADHD/ADD	617	15.7	1,044	5.6
Anxiety/Depression	831	21.1	2,158	11.5
Parental SES				
Low	867	22.0	2,700	14.4
Medium-low	1,540	39.1	6,023	32.1
Medium-high	1,159	29.4	6,587	35.2
High	355	9.0	3,218	17.2
Missing	21	0.5	200	1.1
Maternal education				
Compulsory school	1,582	40.1	5,615	30.0
Upper secondary school	1,541	39.1	7,167	38.3
University degree	730	18.5	5,383	28.7
Missing	89	2.3	563	3.0
Paternal education				
Compulsory school	1,273	32.3	4,402	23.5
Upper secondary school	1,890	48.0	9,095	48.6
University degree	482	12.2	3,912	20.9
Missing	297	7.5	1,319	7.0
Maternal income				
Q1	1,173	29.8	4,887	26.1
Q2	1,281	32.5	5,843	31.2
Q3	931	23.6	4,585	24.5
Q4	486	12.3	2,988	15.9
Missing	71	1.8	425	2.3
Paternal income				
Q1	1,127	28.6	3,917	20.9
Q2	760	19.3	3,128	16.7
Q3	889	22.5	4,389	23.4
Q4	864	21.9	5,986	32.0
Missing	302	7.7	1,308	7.0
Maternal occupational status				
Occupation	2,963	75.2	15,405	82.2
No occupation	916	23.2	2,956	15.8
Missing	63	1.6	367	2.0
Paternal occupational status				
Occupation	2,868	72.8	14,906	79.6
No occupation	800	20.3	2,627	14.0
Missing	274	6.9	1,195	6.4

Data are n % if not else stated. Abbreviation: SES, socioeconomic status; Q, quartile.
*Median with interquartile ranges

For peer review only

Results

In total, 3,942 individuals in the childhood obesity cohort and 18,728 individuals in the comparison group were included in the study (Table 1). In both groups, 46% of the participants were girls and the median age at follow-up was 23.4 years. The proportions of individuals of Nordic origin were similar in both groups (73.7% vs. 75.0%; p=0.02). Despite the groups being matched for living area, a greater proportion of children with obesity grew up in households with low SES compared with the comparison group (22.0% vs. 14.4%; p<0.0001).

Parental SES, childhood obesity and the child's attained level of education

In the childhood obesity cohort, 56.7% completed ≥12 years in school, compared with 74.4% in the comparison group. Girls more frequently completed ≥12 years of schooling than boys in both groups (S2 Table).

Higher parental SES was positively associated with completion of ≥12 years of schooling in both the childhood obesity cohort and in the comparison group (Fig 2). The odds were more than five times higher among children growing up in high SES households compared with those growing up in low SES households (Table 2). However, even when taking parental SES and other risk factors into account, individuals in the childhood obesity cohort were almost half as likely to complete ≥12 school years compared with those in the comparison group (Table 2). The adjusted odds ratio ([a]OR) [99% CI] to complete ≥12 years of schooling stratified by parental SES, when comparing the childhood obesity cohort with the comparison

For peer review only - http://bmjopen.bmj.com/site/about/guidelines.xhtml

group, showed a trend towards lower OR in the higher level of SES: low parental SES = 0.69 [0.50 to 0.95], p=0.0026; medium-low parental SES = 0.59 [0.48 to 0.72], $p<0.0001$; medium-high parental SES = 0.46 [0.35 to 0.60], $p<0.0001$; high parental SES = 0.27 [0.14 to 0.54], $p<0.0001$. P-value for interaction test for childhood obesity and parental SES reaches a p of 0.0015.

The association of parental SES on school performance was more pronounced in the comparison group than in the childhood obesity cohort (S3 Table). For example, stratified analyses show that adjusted OR of completing ≥12 years in school in low SES compared with high SES in the comparison group was 0.17 (99% CI 0.14 to 0.21, $p<0.0001$) and in the childhood obesity cohort 0.31 (99% CI 0.22 to 0.45, $p<0.0001$). Regardless of how we divide the childhood obesity population (into age- or calendar year at start of obesity treatment, SES sub-scores), large differences between the two groups remain (Table 2 and S4 Table).

For peer review only

For peer review only - http://bmjopen.bmj.com/site/about/guidelines.xhtml

Table 2. Crude and adjusted odds ratio (99% CI); p-value of subjects completing ≥12 years of schooling

	Crude estimates	Model 1, n=22 449	Model 2, n=20 610	Model 3, n=20 691	Model 4, n=20 885	Model 5, n=20 420
Childhood obesity cohort vs. comparison group	0.44 (0.40 to 0.48); <0.0001	0.57 (0.51 to 0.63); <0.0001	0.55 (0.50 to 0.62); <0.0001	0.54 (0.48 to 0.61); <0.0001	0.53 (0.48 to 0.59); <0.0001	0.59 (0.52 to 0.66); <0.0001
Non-Nordic vs. Nordic	0.70 (0.63 to 0.78); <0.0001	0.85 (0.75 to 0.97); 0.001	0.77 (0.67 to 0.88); <0.0001	0.87 (0.76 to 0.99); 0.0068	0.81 (0.71 to 0.92); <0.0001	0.97 (0.84 to 1.12); 0.5687
ADHD/ADD vs. non-ADHD/ADD	0.19 (0.17 to 0.23); <0.0001	0.28 (0.24 to 0.33); <0.0001	0.27 (0.22 to 0.33); <0.0001	0.27 (0.22 to 0.32); <0.0001	0.26 (0.22 to 0.31); <0.0001	0.28 (0.23 to 0.34); <0.0001
Anxiety/depression vs. no anxiety/depression	0.31 (0.27 to 0.35); <0.0001	0.39 (0.34 to 0.45); <0.0001	0.36 (0.31 to 0.41); <0.0001	0.38 (0.33 to 0.44); <0.0001	0.39 (0.33 to 0.44); <0.0001	0.36 (0.31 to 0.42); <0.0001
Parental SES						
Medium-low vs. low parental SES	1.74 (1.54 to 1.97); <0.0001	1.69 (1.48 to 1.93); <0.0001				
Medium-high vs. low parental SES	3.33 (2.92 to 3.81); <0.0001	2.99 (2.59 to 3.45); <0.0001				
High vs. low parental SES	6.21 (5.19 to 7.44); <0.0001	5.40 (4.45 to 6.55); <0.0001				
Maternal education						
Upper secondary school vs. compulsory school	1.88 (1.69 to 2.08); <0.0001		1.63 (1.45 to 1.83); <0.0001			1.51 (1.34 to 1.71); <0.0001
University degree vs. compulsory school	3.23 (2.86 to 3.66); <0.0001		2.45 (2.12 to 2.83); <0.0001			2.17 (1.87 to 2.53); <0.0001
Paternal education						
Upper secondary school vs. compulsory school	1.88 (1.69 to 2.09); <0.0001		1.58 (1.40 to 1.77); <0.0001			1.42 (1.26 to 1.60); <0.0001
University degree vs. compulsory school	3.07 (2.66 to 3.55); <0.0001		2.09 (1.77 to 2.45); <0.0001			1.81 (1.53 to 2.15); <0.0001
Maternal income						
Q2 vs. Q1	1.28 (1.15 to 1.43); <0.0001			1.25 (1.10 to 1.41); <0.0001		1.07 (0.93 to 1.23); 0.2065
Q3 vs. Q1	1.45 (1.28 to 1.63); <0.0001			1.35 (1.17 to 1.55); <0.0001		1.05 (0.90 to 1.23); 0.4224
Q4 vs. Q1	1.86 (1.60 to 2.15); <0.0001			1.52 (1.28 to 1.81); <0.0001		1.07 (0.88 to 1.28); 0.3831
Paternal income						
Q2 vs. Q1	1.36 (1.19 to 1.55); <0.0001			1.31 (1.13 to 1.51); <0.0001		1.07 (0.91 to 1.26); 0.2740
Q3 vs. Q1	1.90 (1.67 to 2.17); <0.0001			1.70 (1.48 to 1.96); <0.0001		1.24 (1.05 to 1.46); 0.0009
Q4 vs. Q1	2.95 (2.58 to 3.36); <0.0001			2.47 (2.13 to 2.86); <0.0001		1.58 (1.33 to 1.88); <0.0001
Maternal occupational status						
Occupation vs. no occupation	2.15 (1.92 to 2.40); <0.0001				1.72 (1.51 to 1.96); <0.0001	1.38 (1.19 to 1.61); <0.0001
Paternal occupational status						
Occupation vs. no occupation	2.24 (1.99 to 2.52); <0.0001				1.79 (1.57 to 2.04); <0.0001	1.41 (1.20 to 1.65); <0.0001

Abbreviations: CI, confidence interval; SES, socioeconomic status.
Model 1: Variables included were group (childhood obesity cohort vs. comparison group), migration background, ADHD/ADD, anxiety/depression, and parental SES
Model 2: Variables included were group, migration background, ADHD/ADD, anxiety/depression, maternal-, and paternal education
Model 3: Variables included were group, migration background, ADHD/ADD, anxiety/depression, maternal-, and paternal income
Model 4: Variables included were group, migration background, ADHD/ADD, anxiety/depression, maternal-, and paternal occupational status.
Model 5: Variables included were group, migration background, ADHD/ADD, anxiety/depression, maternal-, and paternal education, income, and occupational status.

For peer review only

For peer review only - http://bmjopen.bmj.com/site/about/guidelines.xhtml

Of note, having a non-Nordic-, compared to a Nordic background, was associated with reduced odds to complete 12 or more years of schooling (Table 2). This was however not observed in boys in the childhood obesity cohort (p=0.68). Further, excluding individuals with a non-Swedish background, or ADHD/ADD, or anxiety/depression in sensitivity analyses, did not alter the association between childhood obesity and attained level of education. For example, when excluding individuals with a non-Swedish background, the [a]OR to complete ≥12 years in school for the obesity cohort vs. the comparison group was 0.55 [0.48 to 0.63], p <0.0001.

Degree of obesity and treatment response on completed educational level in children with obesity

In the childhood obesity cohort, the mean age at start of treatment was 13.5 years (99% CI: 13.45 to 13.62) and the mean BMI SDS was +2.91 (99% CI: 2.89 to 2.92). The median treatment duration was 2.83 years (IQR: 1.86 to 4.42) and the mean treatment response, calculated using BMI SDS from the first to the last clinical visit, was -0.13 (99% CI: -0.15 to -0.10, n=2,709, 68.7%, dropouts excluded). At baseline, 50.7% had obesity and 49.3% had morbid obesity. At last measured weight and height, 33.4% had obesity and 47.2% had morbid obesity, while 19.4% of the children no longer had obesity (dropouts excluded).

A greater degree of obesity at start of treatment lowered the odds of completing ≥12 years of schooling, [a]OR [99% CI] = 0.51 [0.40 to 0.64], p<0.0001, per one unit increase in BMI SDS, while age at start of obesity treatment did not influence the outcome (Table 3). Treatment response was categorized as good response (n=847), no response (n=1 315), poor response (n=547), and dropouts (n=1,233). Of those with good treatment response, 67% completed

For peer review only - http://bmjopen.bmj.com/site/about/guidelines.xhtml

≥12 years in school compared with 58%, 52%, and 50% in the groups with no or poor response, and dropouts, respectively ($p<0.0001$). Within all SES groups, except for high SES, greater treatment response resulted in higher odds of completing ≥12 years in school (Fig 3). Dropouts were less likely to complete ≥12 years in school compared with non-responders (Table 3) but this was not observed in the high SES group (Fig 3). Duration of treatment differed between children with good and poor treatment response (3.5 vs. 4.0 years; $p<0.0001$). In the childhood obesity cohort, the association between parental SES and odds of completing ≥12 years of schooling was not modified by treatment response ($p=0.603$).

For peer review only

For peer review only - http://bmjopen.bmj.com/site/about/guidelines.xhtml

Table 3. Crude and adjusted odds ratios of completing ≥12 years of schooling in the childhood obesity cohort

	**Odds ratio (99% CI) performed with ordinary logistic regression; p value**		
	Crude estimates	Model 1, n=3 921	Model 2, n=3 575
Sex (girls vs. boys)	1.26 (1.06 to 1.48); 0.0004	1.30 (1.08 to 1.56); 0.0002	1.29 (1.06 to 1.56); 0.0008
Migration background (non-Nordic vs. Nordic)	0.78 (0.65 to 0.94); 0.0006	0.74 (0.60 to 0.91); 0.0002	0.69 (0.55 to 0.87); <0.0001
ADHD/ADD (yes vs. no)	0.31 (0.25 to 0.40); <0.0001	0.35 (0.27 to 0.45); <0.0001	0.33 (0.26 to 0.44); <0.0001
Anxiety/depression (yes vs. no)	0.43 (0.35 to 0.53); <0.0001	0.45 (0.36 to 0.57); <0.0001	0.43 (0.34 to 0.55); <0.0001
BMI SDS at start of treatment	0.47 (0.38 to 0.58); <0.0001	0.51 (0.40 to 0.64); <0.0001	0.48 (0.38 to 0.62); <0.0001
Age at start of treatment	0.96 (0.93 to 1.01); 0.0234	1.01 (0.96 to 1.06); 0.60	1.01 (0.96 to 1.06); 0.805
Treatment response*			
Good response vs. no response	1.46 (1.15 to 1.85); <0.0001	1.34 (1.04 to 1.72); 0.0031	1.28 (0.98 to 1.67); 0.0182
Poor response vs. no response	0.78 (0.60 to 1.02); 0.0167	0.72 (0.54 to 0.96); 0.0028	0.71 (0.53 to 0.96); 0.003
Dropouts vs. no response	0.71 (0.58 to 0.87); <0.0001	0.69 (0.55 to 0.86); <0.0001	0.68 (0.54 to 0.87); <0.0001
Parental SES			
Medium-low vs. low parental SES	1.63 (1.31 to 2.03); <0.0001	1.56 (1.24 to 1.98); <0.0001	
Medium-high vs. low parental SES	2.40 (1.90 to 3.05); <0.0001	2.19 (1.69 to 2.83); <0.0001	
High vs. low parental SES	2.99 (2.11 to 4.22); <0.0001	2.83 (1.95 to 4.12); <0.0001	
Maternal education			
Upper secondary school vs. compulsory school	1.74 (1.44 to 2.09); <0.0001		1.57 (1.27 to 1.94); <0.0001
University degree vs. compulsory school	2.47 (1.94 to 3.16); <0.0001		2.23 (1.67 to 2.98); <0.0001
Paternal education			
Upper secondary school vs. compulsory school	1.48 (1.23 to 1.79); <0.0001		1.26 (1.03 to 1.55); 0.004
University degree vs. compulsory school	1.92 (1.44 to 2.56); <0.0001		1.41 (1.01 to 1.95); 0.0076

Abbreviations: CI, confidence interval; BMI SDS, BMI standard deviation score; SES, socioeconomic status.
* Good response=decrease of BMI SDS ≥ 0.25 units; No response=BMI SDS +/- 0.24 units; Poor response=increase of BMI SDS ≥ 0.25 units; Dropouts=no follow-up measure or less than one year between their first and last measure.
Model 1: Variables included were sex, migration background, ADHD/ADD, anxiety/depression, BMI SDS- and age at start of treatment, treatment response, and parental SES.
Model 2: Variables included were sex, migration background, ADHD/ADD, anxiety/depression, BMI SDS- and age at start of treatment, treatment response, maternal-, and paternal education.

For peer review only

Discussion

In this prospective cohort study, we have compared level of education among individuals recorded in the Swedish Childhood Obesity Treatment Register, and a comparison group matched by sex, year of birth, and living area. Individuals in the childhood obesity cohort were half as likely to complete 12 or more years of schooling, independently of parental socioeconomic status (SES).

Among individuals from high SES families, those in the childhood obesity cohort were approximately one third as likely to complete ≥12 years of schooling as individuals from high SES families in the comparison group. Furthermore, our results indicate that parental SES was more important to complete ≥12 school years in the comparison group than in the childhood obesity cohort. An association between obesity and impaired academic achievements has been demonstrated before.[8,9] To which extent other psychosocial factors contribute to this finding has not been evaluated. It is well established that low parental SES,[13-15] immigrant background,[9,14] ADHD/ADD,[9,17] and depression,[9,14,16] may negatively affect performance in school. These factors are also overrepresented in the paediatric population with obesity. In this study, we can confirm that low parental SES, neuropsychiatric disorders, and anxiety/depression are more common in children with obesity and contribute to the decreased odds of completing ≥12 school years. However, we found that obesity in childhood is a considerable risk factor for not completing ≥12 years of schooling even after taking these and other important risk factors into account.

Obesity treatment outcome and educational level

For peer review only

For peer review only - http://bmjopen.bmj.com/site/about/guidelines.xhtml

We identified a positive association between weight-loss during obesity treatment and completed educational level, which confirms our previously reported data unadjusted for SES.[9] Children with good treatment response, compared with those with no response, were more likely to complete ≥12 school years in all SES groups. A decrease of 0.25 BMI SDS, that has previously been shown to improve metabolic health,[27] we can now also show may have a positive association on completing ≥12 years of schooling. However, the positive effects of successful obesity treatment did not compensate for the observed SES differences on attained level of education.

In Sweden, 12 years of schooling is not mandatory, but the long-term social effects are considerable for those who fail. It has been estimated that 50% of those who fail to complete ≥12 school years face a situation of being left outside the society with poor psychosocial health and large costs for the society.[28] Thus, it is possible that school failure will further worsen the health perspective of adolescents with obesity.

Potential mechanisms between obesity, weight loss, and school performance

Several parameters may interplay when measuring school completion, e.g. resourcefulness, intelligence, or ability to conform. Nevertheless, the mechanisms by which obesity influences school performance are unclear but are likely multifactorial and complex. Possible mechanisms may include psychosocial aspects such as stigma[29] and increased risk for anxiety and depression.[7] Physiological mechanisms may be mediated by anatomical changes such as atrophy of the frontal lobe and reduced hippocampal volume have been demonstrated in individuals with obesity.[30] Some of the reported cognitive dysfunctions in children and adolescents with obesity include slower response times when performing visuospatial attention tasks,[31] diminished executive functions, such as working memory,[32] and slower cognitive

For peer review only - http://bmjopen.bmj.com/site/about/guidelines.xhtml

performance speed.[33] In addition, obesity-related comorbidities such as insulin resistance,[34] type 2 diabetes mellitus,[35] chronic low-grade inflammation,[36] and the metabolic syndrome[37] have also been associated with impaired executive function, memory performance, attention, and cognitive flexibility. A high fat diet in rodents demonstrated detrimental effects on memory and executive functions.[30]

At least some of these effects seem to be reversible, which may add to the positive associations we observed of weight loss on school outcome. Weight loss may have a direct positive effect on cognitive functions.[38] Extensive weight loss via bariatric surgery improves insulin sensitivity and decreases systematic inflammation,[39] and it has been suggested that these factors affect cognitive functions.[40]

However, children with obesity are often stigmatized,[41] have a low self-esteem, and are exposed to bullying and social exclusion.[42] All these factors have most likely a negative impact on school performance. In contrast, a strong social network is most probably an important factor both for good treatment response and achievement in school. An inverse association has been observed between familial social support and child weight status.[43] The relationship between BMI and school completion may also be biased from e.g. assortative mating and dynastic effects which have shown to reduce causal effects.[44] There is also a suggested interplay between genetic variants and environmental factors that may affect intelligence.[45] Thus, it is likely that both negative social effects of obesity and obesity-related morbidity, as well as genetic factors, contribute to the adverse association of childhood obesity on completed educational level.[7 29 41 45]

Limitations

Using longitudinal data from several national registers provided an opportunity to assess the impact of obesity on completed educational level, adjusted for several important confounders.

For peer review only - http://bmjopen.bmj.com/site/about/guidelines.xhtml

Data on both exposure and outcome were prospectively collected and defined according to standardized international classifications.[19 26]

However, some important limitations should be recognized. We did not have anthropometric data on children in the comparison group. There is no representative national data on children with obesity in Sweden. As our comparison group includes children with obesity, although likely less than 1% according to obesity diagnoses found in the National Patient Register, odds of lower level of education associated with obesity might be underestimated. It is also important to consider that children receiving obesity treatment may not be representative of all children with obesity. It should further be noted that parental SES was based on data from one specific year in the child's life and not over the child's entire adolescent lifetime, and that the SES indicators used may not reflect the whole SES spectrum. After providing specific ORs for each SES indicator (Table 2) we can hypothesize that additive, or perhaps synergistic effects, of different SES indicators may affect the outcome. Nevertheless, the OR for the childhood obesity cohort vs. comparison group remained within a narrow interval. This may indicate that there is an overlap between the measured SES domains and/or that the effect of obesity is a robust variable, independently of SES. In addition, the impact of anxiety and depression on educational level may be underestimated since these conditions often are under-diagnosed. It is furthermore plausible that controlling for anxiety and depression may lead to an underestimation of the association found between obesity in childhood and completion of 12 or more school years, since the variable may act as a modifier/mediator.[7] We urge the reader to bear in mind that despite several possible mechanisms have been proposed, causal relationships of obesity and the effect of treatment on the outcome remains to be established. Lastly, factors such as free education, school lunches, and students' health care may have an impact on the generalizability of our data to other populations.

Conclusion

For peer review only - http://bmjopen.bmj.com/site/about/guidelines.xhtml

In this longitudinal, population-based study, individuals with obesity in childhood were less likely to complete 12 or more years of schooling, compared with a group from the general population. The odds associated with obesity remained significantly increased even after taking parental SES and other important risk factors, such as ADHD, into consideration. The underlying mechanisms are unclear but previous studies indicate that the effects of obesity on school completion both involve psychosocial effects and cognitive functions.[16 17 32] The negative impact of childhood obesity on educational level may partly be reversed by successful obesity treatment in childhood. Results from this study underline effects of childhood obesity on schooling. For the sake of an optimal educational environment, it is of great importance to increase awareness both in schools and among decision makers to allocate resources for potential extra support, e.g. reduce stigma and increase educational support.

Acknowledgements

The authors would like to thank all local health care professionals in Sweden working with children and adolescents with obesity and their valuable work with BORIS, and the BORIS steering committee for establishing and maintaining the register.

For peer review only

Author contributions

Conceptualization: Louise Lindberg, Pernilla Danielsson, Martina Persson, Claude Marcus, Emilia Hagman.

Data curation: Louise Lindberg.

Formal analysis: Louise Lindberg, Emilia Hagman.

Funding acquisition: Louise Lindberg, Claude Marcus, Emilia Hagman.

Methodology: Louise Lindberg, Emilia Hagman.

Project administration: Louise Lindberg, Emilia Hagman.

Supervision: Martina Persson, Emilia Hagman, Pernilla Danielsson, Claude Marcus.

Writing – original draft: Louise Lindberg.

For peer review only - http://bmjopen.bmj.com/site/about/guidelines.xhtml

Writing – review & editing: Louise Lindberg, Pernilla Danielsson, Martina Persson, Claude Marcus, Emilia Hagman.

Funding statement

This study was supported by funds to LL by Crown Princess Lovisa's Foundation (2017-00348), Samariten Foundation (2017-0305), the Stockholm FreeMason Foundation for Children's Welfare, Sällskapet Barnavård, Anna-Lisa och Arne Gustafssons Foundation, Solstickan Foundation and Sven Jerring Foundation. EH was supported by the Swedish Society for Medical Research, Fredrik and Ingrid Thuring's Foundation (2017-00309) and Magnus Bergvall Foundation (2017-02113), and CM by the Swedish Heart and Lung Foundation (20150790).

Data Sharing statement

The data that support the findings of this study contains sensitive information. Restrictions therefore apply to the availability of these data, which were used under license for the current study, and so are not publicly available. According to Swedish law and the General Data Protection Regulation, the authors are not permitted to share the datasets used in this study with third parties. Given that an ethical approval is obtained, any individual may apply for data from Statistics Sweden via information@scb.se, the Swedish National Board of Health and Welfare via registerservice@socialstyrelsen.se, and the Swedish Childhood Obesity Treatment Register via http://www.e-boris.se/kontaktuppgifter/.

Competing interests

All authors have completed the ICMJE uniform disclosure form at www.icmje.org/coi_disclosure.pdf and declare: no support from any organisation for the submitted work; no financial relationships with any organisations that might have an interest in

For peer review only - http://bmjopen.bmj.com/site/about/guidelines.xhtml

the submitted work in the previous three years; no other relationships or activities that could appear to have influenced the submitted.

For peer review only

For peer review only - http://bmjopen.bmj.com/site/about/guidelines.xhtml

References

1. Abarca-Gómez L, Abdeen ZA, Hamid ZA, et al. Worldwide trends in body-mass index, underweight, overweight, and obesity from 1975 to 2016: a pooled analysis of 2416 population-based measurement studies in 128.9 million children, adolescents, and adults. *The Lancet* 2017 doi: 10.1016/S0140-6736(17)32129-3
2. Hagman E, Danielsson P, Brandt L, et al. Association between impaired fasting glycaemia in pediatric obesity and type 2 diabetes in young adulthood. *Nutr Diabetes* 2016;6(8):e227. doi: 10.1038/nutd.2016.34 [published Online First: 2016/08/23]
3. Hagman E, Danielsson P, Elimam A, et al. The effect of weight loss and weight gain on blood pressure in children and adolescents with obesity. *Int J Obes (Lond)* 2019;43(10):1988-94. doi: 10.1038/s41366-019-0384-2 [published Online First: 2019/06/04]
4. Lindberg L, Danielsson P, Persson M, et al. Association of childhood obesity with risk of early all-cause and cause-specific mortality: A Swedish prospective cohort study. *PLoS medicine* 2020;17(3):e1003078. doi: 10.1371/journal.pmed.1003078 [published Online First: 2020/03/19]
5. Reilly JJ, Kelly J. Long-term impact of overweight and obesity in childhood and adolescence on morbidity and premature mortality in adulthood: systematic review. *Int J Obes (Lond)* 2011;35(7):891-8. doi: 10.1038/ijo.2010.222
6. Viner RM, Cole TJ. Adult socioeconomic, educational, social, and psychological outcomes of childhood obesity: a national birth cohort study. *BMJ* 2005;330(7504):1354. doi: 10.1136/bmj.38453.422049.E0 [published Online First: 2005/05/20]
7. Lindberg L, Hagman E, Danielsson P, et al. Anxiety and depression in children and adolescents with obesity: a nationwide study in Sweden. *BMC medicine* 2020;18(1):30. doi: 10.1186/s12916-020-1498-z [published Online First: 2020/02/23]
8. Laitinen J, Power C, Ek E, et al. Unemployment and obesity among young adults in a northern Finland 1966 birth cohort. *International journal of obesity and related metabolic disorders : journal of the International Association for the Study of Obesity* 2002;26(10):1329-38. doi: 10.1038/sj.ijo.0802134
9. Hagman E, Danielsson P, Brandt L, et al. Childhood Obesity, Obesity Treatment Outcome, and Achieved Education: A Prospective Cohort Study. *The Journal of adolescent health : official publication of the Society for Adolescent Medicine* 2017 doi: 10.1016/j.jadohealth.2017.04.009 [published Online First: 2017/07/12]
10. Karnehed N, Rasmussen F, Hemmingsson T, et al. Obesity and attained education: cohort study of more than 700,000 Swedish men. *Obesity* 2006;14(8):1421-28.
11. French SA, Wall M, Corbeil T, et al. Obesity in Adolescence Predicts Lower Educational Attainment and Income in Adulthood: The Project EAT Longitudinal Study. *Obesity (Silver Spring)* 2018;26(9):1467-73. doi: 10.1002/oby.22273 [published Online First: 2018/09/19]
12. Shrewsbury V, Wardle J. Socioeconomic status and adiposity in childhood: a systematic review of cross-sectional studies 1990-2005. *Obesity (Silver Spring)* 2008;16(2):275-84. doi: 10.1038/oby.2007.35 [published Online First: 2008/02/02]
13. Koivusilta LK, West P, Saaristo VM, et al. From childhood socio-economic position to adult educational level - do health behaviours in adolescence matter? A longitudinal study. *BMC public health* 2013;13:711. doi: 10.1186/1471-2458-13-711
14. Kark M, Hjern A, Rasmussen F. Poor school performance is associated with a larger gain in body mass index during puberty. *Acta Paediatr* 2014;103(2):207-13. doi: 10.1111/apa.12471
15. DeGarmo DS, Forgatch MS, Martinez CR, Jr. Parenting of divorced mothers as a link between social status and boys' academic outcomes: unpacking the effects of socioeconomic status. *Child development* 1999;70(5):1231-45. [published Online First: 1999/11/05]

For peer review only

For peer review only - http://bmjopen.bmj.com/site/about/guidelines.xhtml

16. Freeman A, Tyrovolas S, Koyanagi A, et al. The role of socio-economic status in depression: results from the COURAGE (aging survey in Europe). *BMC public health* 2016;16(1):1098. doi: 10.1186/s12889-016-3638-0 [published Online First: 2016/10/21]
17. Scholtens S, Rydell AM, Yang-Wallentin F. ADHD symptoms, academic achievement, self-perception of academic competence and future orientation: a longitudinal study. *Scandinavian journal of psychology* 2013;54(3):205-12. doi: 10.1111/sjop.12042 [published Online First: 2013/03/21]
18. Quek YH, Tam WWS, Zhang MWB, et al. Exploring the association between childhood and adolescent obesity and depression: a meta-analysis. *Obesity reviews : an official journal of the International Association for the Study of Obesity* 2017;18(7):742-54. doi: 10.1111/obr.12535 [published Online First: 2017/04/13]
19. Cole TJ, Lobstein T. Extended international (IOTF) body mass index cut-offs for thinness, overweight and obesity. *Pediatr Obes* 2012;7(4):284-94. doi: 10.1111/j.2047-6310.2012.00064.x
20. Hagman E, Danielsson P, Lindberg L, et al. Paediatric obesity treatment during 14 years in Sweden: Lessons from the Swedish Childhood Obesity Treatment Register-BORIS. *Pediatr Obes* 2020;15(7):e12626. doi: 10.1111/ijpo.12626 [published Online First: 2020/02/20]
21. Ludvigsson JF, Otterblad-Olausson P, Pettersson BU, et al. The Swedish personal identity number: possibilities and pitfalls in healthcare and medical research. *European journal of epidemiology* 2009;24(11):659-67. doi: 10.1007/s10654-009-9350-y
22. Ludvigsson JF, Almqvist C, Bonamy AK, et al. Registers of the Swedish total population and their use in medical research. *European journal of epidemiology* 2016;31(2):125-36. doi: 10.1007/s10654-016-0117-y [published Online First: 2016/01/16]
23. Ludvigsson JF, Andersson E, Ekbom A, et al. External review and validation of the Swedish national inpatient register. *BMC public health* 2011;11:450. doi: 10.1186/1471-2458-11-450 [published Online First: 2011/06/11]
24. Wallerstedt SM, Wettermark B, Hoffmann M. The First Decade with the Swedish Prescribed Drug Register - A Systematic Review of the Output in the Scientific Literature. *Basic & clinical pharmacology & toxicology* 2016;119(5):464-69. doi: 10.1111/bcpt.12613 [published Online First: 2016/04/27]
25. Brooke HL, Talback M, Hornblad J, et al. The Swedish cause of death register. *European journal of epidemiology* 2017;32(9):765-73. doi: 10.1007/s10654-017-0316-1 [published Online First: 2017/10/07]
26. UNESCO. International standard classification of education: Isced 1997. Re-edition. Paris: UNESCO Institute for Statistics. 2011
27. Ford AL, Hunt LP, Cooper A, et al. What reduction in BMI SDS is required in obese adolescents to improve body composition and cardiometabolic health? *Archives of disease in childhood* 2010;95(4):256-61. doi: 10.1136/adc.2009.165340 [published Online First: 2009/12/08]
28. Nilsson I, Wadeskog A. Utanförskapets Ekonomiska Sociotoper: Socioekonomisk analys på stadsdelsnivå inom ramen för Healthy Cities 2012
29. Finn KE, Seymour CM, Phillips AE. Weight bias and grading among middle and high school teachers. *Br J Educ Psychol* 2019 doi: 10.1111/bjep.12322 [published Online First: 2019/10/28]
30. Nguyen JC, Killcross AS, Jenkins TA. Obesity and cognitive decline: role of inflammation and vascular changes. *Frontiers in neuroscience* 2014;8:375. doi: 10.3389/fnins.2014.00375 [published Online First: 2014/12/06]
31. Tsai C-L, Chen F-C, Pan C-Y, et al. The neurocognitive performance of visuospatial attention in children with obesity. *Frontiers in psychology* 2016;7
32. Laurent JS, Watts R, Adise S, et al. Associations Among Body Mass Index, Cortical Thickness, and Executive Function in Children. *JAMA Pediatr* 2019 doi: 10.1001/jamapediatrics.2019.4708 [published Online First: 2019/12/10]

For peer review only - http://bmjopen.bmj.com/site/about/guidelines.xhtml

33. Sweat V, Yates KF, Migliaccio R, et al. Obese adolescents show reduced cognitive processing speed compared with healthy weight peers. *Childhood Obesity* 2017

34. Cheke LG, Bonnici HM, Clayton NS, et al. Obesity and insulin resistance are associated with reduced activity in core memory regions of the brain. *Neuropsychologia* 2017;96:137-49.

35. Bruehl H, Sweat V, Tirsi A, et al. Obese Adolescents with Type 2 Diabetes Mellitus Have Hippocampal and Frontal Lobe Volume Reductions. *Neuroscience and medicine* 2011;2(1):34-42. doi: 10.4236/nm.2011.21005

36. Spyridaki EC, Simos P, Avgoustinaki PD, et al. The association between obesity and fluid intelligence impairment is mediated by chronic low-grade inflammation. *British Journal of Nutrition* 2014;112(10):1724-34.

37. Mangone A, Yates KF, Sweat V, et al. Cognitive functions among predominantly minority urban adolescents with metabolic syndrome. *Applied Neuropsychology: Child* 2017:1-7.

38. Alosco ML, Spitznagel MB, Strain G, et al. Improved memory function two years after bariatric surgery. *Obesity* 2014;22(1):32-38.

39. Olbers T, Beamish AJ, Gronowitz E, et al. Laparoscopic Roux-en-Y gastric bypass in adolescents with severe obesity (AMOS): a prospective, 5-year, Swedish nationwide study. *Lancet Diabetes Endocrinol* 2017;5(3):174-83. doi: 10.1016/S2213-8587(16)30424-7

40. Biessels GJ, Reagan LP. Hippocampal insulin resistance and cognitive dysfunction. *Nature Reviews Neuroscience* 2015;16(11):660.

41. Latner JD, Stunkard AJ. Getting worse: the stigmatization of obese children. *Obesity research* 2003;11(3):452-6. doi: 10.1038/oby.2003.61

42. Pierce JW, Wardle J. Cause and effect beliefs and self-esteem of overweight children. *Journal of child psychology and psychiatry, and allied disciplines* 1997;38(6):645-50. [published Online First: 1997/10/08]

43. Lindberg L, Ek A, Nyman J, et al. Low grandparental social support combined with low parental socioeconomic status is closely associated with obesity in preschool-aged children: a pilot study. *Pediatr Obes* 2015 doi: 10.1111/ijpo.12049

44. Brumpton B, Sanderson E, Heilbron K, et al. Avoiding dynastic, assortative mating, and population stratification biases in Mendelian randomization through within-family analyses. *Nature communications* 2020;11(1):3519. doi: 10.1038/s41467-020-17117-4 [published Online First: 2020/07/16]

45. Plomin R, von Stumm S. The new genetics of intelligence. *Nature reviews Genetics* 2018;19(3):148-59. doi: 10.1038/nrg.2017.104 [published Online First: 2018/01/18]

For peer review only

For peer review only - http://bmjopen.bmj.com/site/about/guidelines.xhtml

Supporting information

S1 Table. International Classification of Diseases (ICD 10th revision) codes and Anatomical Therapeutic Chemical (ATC) classification system codes used.

S2 Table. Proportion of children completing ≥12 years of schooling.

S3 Table. Adjusted odds ratio (99% CI); p-value of subjects completing ≥12 years of schooling stratified by group.

S4 Table. Proportion of individuals who have undergone obesity treatment in childhood who completed ≥12 years of schooling by calendar year and by age at start of obesity treatment (n=3,942).

Figure Legends

Fig 1. Participant flowchart.

Fig 2. Percentage of individuals completing ≥12 years of schooling by SES level in the childhood obesity cohort (n=3,921,) and the comparison group (n=18,528).

Fig 3. Crude percentage of individuals with obesity in childhood (n=3,921) completing ≥12 school years by parental SES and treatment response. p=p for trend; n=refers to number of individuals in each category.

For peer review only - http://bmjopen.bmj.com/site/about/guidelines.xhtml

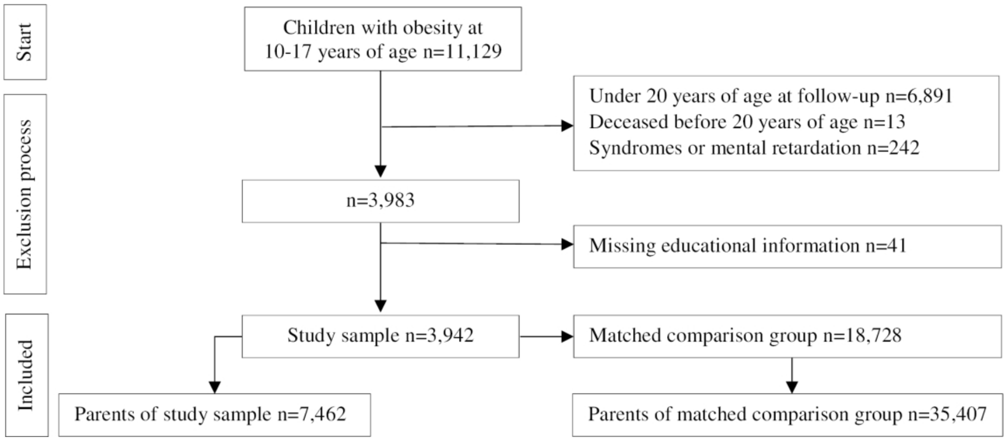

Figure 1

169x73mm (300 x 300 DPI)

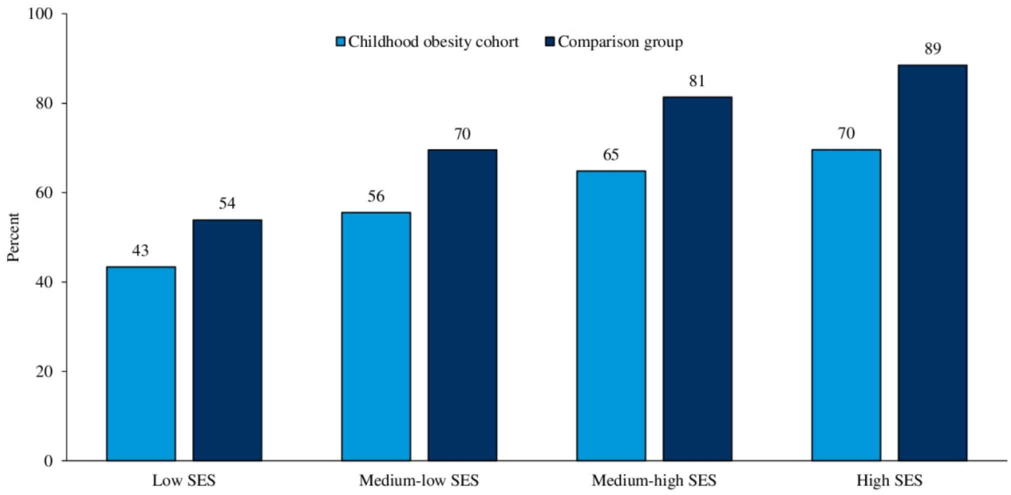

Fig 2. Percentage of individuals completing ≥12 years of schooling by SES level in the childhood obesity cohort (n=3,921) and the comparison group (n=18,528).

118x57mm (300 x 300 DPI)

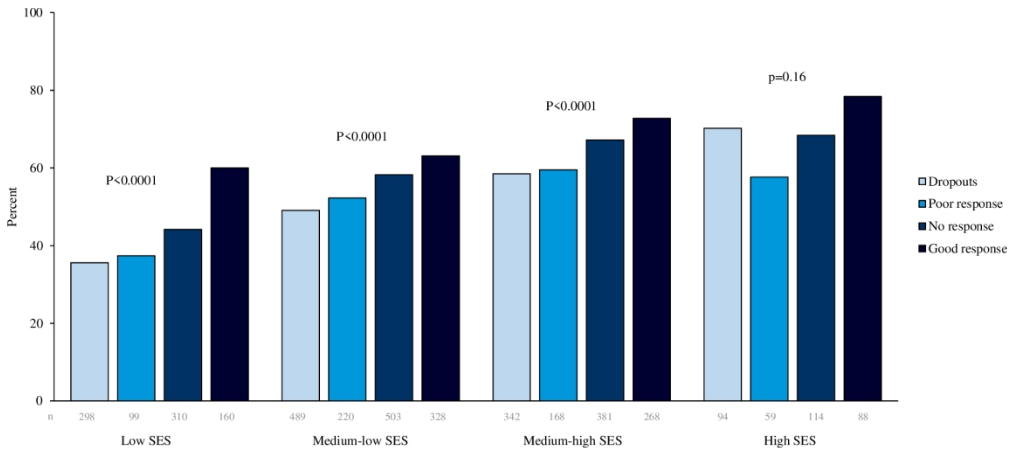

Fig 3. Crude percentage of individuals with obesity in childhood (n=3,921) completing ≥12 school years by parental SES and treatment response. p=p for trend; n=refers to number of individuals in each category.

150x66mm (300 x 300 DPI)

S1 Table. International Classification of Diseases (ICD 10th revision) codes and Anatomical Therapeutic Chemical (ATC) classification system codes used.

Condition	ICD-10	ATC Code
Anxiety/sedatives	F40.0-F40.1, F41-F42, F51.4-F51.9, F93.0-F93.2	N05B
Depression/antidepressants	F32-F39, F92	N06A
ADHD/ADD	F90	N06B
Mental retardation	F70-F79	
Genetic syndromes		
Fragile X	Q99.2	
Klinefelter	Q98	
Laurence-Moon-Bardet-Biedl	Q87.8B	
Mb Down	Q90	
Noonan	Q87.1E	
Prader-Willi	Q87.1F	
Silver Russell	Q87.1G	
Turner	Q96	

For peer review only

S2 Table. Proportion of children completing ≥12 years of schooling

	Childhood obesity cohort		Comparison group		
	n	%	n	%	p-value[¶]
Total	2,236	56.7	13,939	74.4	<0.0001
Sex					
Girl	1,090	59.7	6,843	78.7	<0.0001
Boy	1,146	54.1	7,096	70.8	<0.0001
Migration background					
Nordic	1,695	58.4	10,974	78.1	<0.0001
Non-Nordic	541	52.2	2,965	63.4	<0.0001
ADHD/ADD					
Non-ADHD/ADD	2,032	61.1	13,528	76.5	<0.0001
ADHD/ADD	204	33.1	411	39.4	0.01
Anxiety/depression					
No anxiety/depression	1,902	61.1	12,754	77.0	<0.0001
Anxiety/depression	334	40.2	1,185	54.9	<0.0001
Parental SES					
Low	376	43.4	1,455	53.9	<0.0001
Medium-low	855	55.5	4,187	69.5	<0.0001
Medium-high	751	64.8	5,358	81.3	<0.0001
High	247	69.6	2,848	88.5	<0.0001
Maternal education					
Compulsory school	755	47.7	3,474	61.9	<0.0001
Upper secondary school	945	61.3	5,582	77.9	<0.0001
University degree	506	69.3	4,589	85.3	<0.0001
Paternal education					
Compulsory school	639	50.2	2,775	63.0	<0.0001
Upper secondary school	1,132	59.9	7,085	77.9	<0.0001
University degree	318	66.0	3,333	85.2	<0.0001
Maternal income					
Q1	614	52.3	3,327	68.1	<0.0001
Q2	750	58.6	4,391	75.2	<0.0001
Q3	542	58.2	3,534	77.1	<0.0001
Q4	304	62.6	2,429	81.3	<0.0001
Paternal income					
Q1	556	49.3	2,453	62.6	<0.0001
Q2	410	54.0	2,260	72.3	<0.0001
Q3	549	61.8	3,404	77.6	<0.0001
Q4	572	66.2	5,056	84.5	<0.0001
Maternal occupational status					
No occupation	438	47.8	1,722	58.3	<0.0001
Occupation	1,777	60.0	12,005	77.9	<0.0001
Paternal occupational status					
No occupation	363	45.4	1,543	58.7	<0.0001
Occupation	1,736	60.5	11,703	78.5	<0.0001

Abbreviations: SES, socioeconomic status; Q, quartile. [¶]Performed with chi-square.
1 EUR ≈ 9.83 SEK December 31st 2017. Q1: <18,652 EUR; Q2: 18,652 to 25,025 EUR; Q3: 25,026 to 32,309 EUR; Q4: >32,309 EUR.

For peer review only

S3 Table. Adjusted odds ratio (99% CI); p-value of subjects completing ≥12 years of schooling stratified by group

	Childhood obesity cohort	Comparison group
Parental SES (ref=low)[a]		
Medium-low	1.64 (1.30 to 2.07); <0.0001	1.81 (1.59 to 2.07); <0.0001
Medium-high	2.43 (1.88 to 3.12); <0.0001	3.25 (2.82 to 3.75); <0.0001
High	3.22 (2.24 to 4.64); <0.0001	5.80 (4.81 to 6.99); <0.0001
Maternal education (ref=compulsory school)[b]		
Upper secondary school	1.64 (1.33 to 2.01); <0.0001	1.75 (1.56 to 1.96); <0.0001
University degree	2.33 (1.76 to 3.09); <0.0001	2.54 (2.20 to 2.93); <0.0001
Paternal education (ref=compulsory school)[b]		
Upper secondary school	1.29 (1.05 to 1.58); 0.0012	1.59 (1.42 to 1.79); <0.0001
University degree	1.46 (1.06 to 2.01); 0.0025	2.28 (1.94 to 2.67); <0.0001
Maternal income (ref=Q1)[c]		
Q2	1.28 (1.01 to 1.61); 0.0064	1.26 (1.11 to 1.42); <0.0001
Q3	1.26 (0.98 to 1.62); 0.019	1.30 (1.13 to 1.49); <0.0001
Q4	1.42 (1.04 to 1.95); 0.004	1.47 (1.25 to 1.73); <0.0001
Paternal income (ref=Q1)[c]		
Q2	1.19 (0.92 to 1.54); 0.075	1.44 (1.25 to 1.66); <0.0001
Q3	1.57 (1.22 to 2.02); <0.0001	1.79 (1.56 to 2.05); <0.0001
Q4	1.96 (1.51 to 2.55); <0.0001	2.63 (2.29 to 3.03); <0.0001
Maternal occupational status (ref=no occupation)[d]		
Occupation	1.42 (1.14 to 1.77); <0.0001	1.83 (1.61 to 2.08); <0.0001
Paternal occupational status (ref=no occupation)[d]		
Occupation	1.63 (1.30 to 2.04); <0.0001	1.90 (1.67 to 2.16); <0.0001

[a]Adjusted for migration background, ADHD/ADD, anxiety/depression, parental SES

[b]Adjusted for migration background, ADHD/ADD, anxiety/depression, maternal-, and paternal education

[c]Adjusted for migration background, ADHD/ADD, anxiety/depression, maternal-, and paternal income

[d]Adjusted for migration background, ADHD/ADD, anxiety/depression, maternal-, and paternal occupational status

Abbreviations: SES, socioeconomic status; Q, quartile.
Q1: <18,652 EUR; Q2: 18,652 to 25,025 EUR; Q3: 25,026 to 32,309 EUR; Q4: >32,309 EUR. 1 EUR ≈ 9.83 SEK December 31st 2017.

For peer review only

S4 Table. Proportion of individuals who have undergone obesity treatment in childhood who completed ≥12 years of schooling by calendar year and by age at start of obesity treatment (n=3,942).

Individuals completing ≥12 years of schooling	n	%
Calendar year at start of obesity treatment		
1995-2001	229	62.6
2002-2008	1,262	58.1
2009-2014	745	53.1
Age at start of obesity treatment		
10 - 12.99	960	59.0
13 - 16.99	1,276	55.1

For peer review only

STROBE Statement—Checklist of items that should be included in reports of ***cohort studies***

	Item No	Recommendation	Page No
**Title and abstract**	1	(*a*) Indicate the study's design with a commonly used term in the title or the abstract	1-2
		(*b*) Provide in the abstract an informative and balanced summary of what was done and what was found	2-3
**Introduction**			
Background/rationale	2	Explain the scientific background and rationale for the investigation being reported	4
Objectives	3	State specific objectives, including any prespecified hypotheses	4.5
**Methods**			
Study design	4	Present key elements of study design early in the paper	5
Setting	5	Describe the setting, locations, and relevant dates, including periods of recruitment, exposure, follow-up, and data collection	5-9
Participants	6	(*a*) Give the eligibility criteria, and the sources and methods of selection of participants. Describe methods of follow-up	5
		(*b*) For matched studies, give matching criteria and number of exposed and unexposed	5
Variables	7	Clearly define all outcomes, exposures, predictors, potential confounders, and effect modifiers. Give diagnostic criteria, if applicable	7-9
Data sources/ measurement	8*	For each variable of interest, give sources of data and details of methods of assessment (measurement). Describe comparability of assessment methods if there is more than one group	7-9
Bias	9	Describe any efforts to address potential sources of bias	7-9
Study size	10	Explain how the study size was arrived at	5
Quantitative variables	11	Explain how quantitative variables were handled in the analyses. If applicable, describe which groupings were chosen and why	9-10
Statistical methods	12	(*a*) Describe all statistical methods, including those used to control for confounding	9-10
		(*b*) Describe any methods used to examine subgroups and interactions	
		(*c*) Explain how missing data were addressed	
		(*d*) If applicable, explain how loss to follow-up was addressed	
		(*e*) Describe any sensitivity analyses	
**Results**			
Participants	13*	(a) Report numbers of individuals at each stage of study—eg numbers potentially eligible, examined for eligibility, confirmed eligible, included in the study, completing follow-up, and analysed	12 + fig
		(b) Give reasons for non-participation at each stage	
		(c) Consider use of a flow diagram	
Descriptive data	14*	(a) Give characteristics of study participants (eg demographic, clinical, social) and information on exposures and potential confounders	11
		(b) Indicate number of participants with missing data for each variable of interest	
		(c) Summarise follow-up time (eg, average and total amount)	
Outcome data	15*	Report numbers of outcome events or summary measures over time	12

For peer review only

Main results	16	(*a*) Give unadjusted estimates and, if applicable, confounder-adjusted estimates and their precision (eg, 95% confidence interval). Make clear which confounders were adjusted for and why they were included<br>(*b*) Report category boundaries when continuous variables were categorized<br>(*c*) If relevant, consider translating estimates of relative risk into absolute risk for a meaningful time period	12-17
Other analyses	17	Report other analyses done—eg analyses of subgroups and interactions, and sensitivity analyses	12-17
**Discussion**			
Key results	18	Summarise key results with reference to study objectives	18
Limitations	19	Discuss limitations of the study, taking into account sources of potential bias or imprecision. Discuss both direction and magnitude of any potential bias	20-21
Interpretation	20	Give a cautious overall interpretation of results considering objectives, limitations, multiplicity of analyses, results from similar studies, and other relevant evidence	18-21
Generalisability	21	Discuss the generalisability (external validity) of the study results	21
**Other information**			
Funding	22	Give the source of funding and the role of the funders for the present study and, if applicable, for the original study on which the present article is based	22-23

*Give information separately for exposed and unexposed groups.

Note: An Explanation and Elaboration article discusses each checklist item and gives methodological background and published examples of transparent reporting. The STROBE checklist is best used in conjunction with this article (freely available on the Web sites of PLoS Medicine at http://www.plosmedicine.org/, Annals of Internal Medicine at http://www.annals.org/, and Epidemiology at http://www.epidem.com/). Information on the STROBE Initiative is available at http://www.strobe-statement.org.

For peer review only - http://bmjopen.bmj.com/site/about/guidelines.xhtml
